# Retinal ischemia induces α-SMA-mediated capillary pericyte contraction coincident with perivascular glycogen depletion

**DOI:** 10.1186/s40478-019-0761-z

**Published:** 2019-08-20

**Authors:** Luis Alarcon-Martinez, Sinem Yilmaz-Ozcan, Muge Yemisci, Jesse Schallek, Kıvılcım Kılıç, Deborah Villafranca-Baughman, Alp Can, Adriana Di Polo, Turgay Dalkara

**Affiliations:** 1 Institute of Neurological Sciences and Psychiatry, Hacettepe University, Sihhiye, 06100 Ankara, Turkey; 2 Department of Neurology, Faculty of Medicine, Hacettepe University, Ankara, Turkey; 3 Flaum Eye Institute and Center for Visual Science, University of Rochester, Rochester, New York USA; 4 Department of Histology and Embryology, School of Medicine, Ankara University, Ankara, Turkey; 5 Department of Neuroscience and Centre de Recherche du Centre Hospitalier de l’Université de Montréal, Université de Montréal, Montréal, Québec Canada

**Keywords:** Retinal ischemia/reperfusion, Retinal pericytes, Capillary constriction, Retinal vasculature, Alpha-smooth muscle actin

## Abstract

Increasing evidence indicates that pericytes are vulnerable cells, playing pathophysiological roles in various neurodegenerative processes. Microvascular pericytes contract during cerebral and coronary ischemia and do not relax after re-opening of the occluded artery, causing incomplete reperfusion. However, the cellular mechanisms underlying ischemia-induced pericyte contraction, its delayed emergence, and whether it is pharmacologically reversible are unclear. Here, we investigate *i)* whether ischemia-induced pericyte contractions are mediated by alpha-smooth muscle actin (α-SMA), *ii)* the sources of calcium rise in ischemic pericytes, and *iii)* if peri-microvascular glycogen can support pericyte metabolism during ischemia. Thus, we examined pericyte contractility in response to retinal ischemia both in vivo, using adaptive optics scanning light ophthalmoscopy and, ex vivo, using an unbiased stereological approach. We found that microvascular constrictions were associated with increased calcium in pericytes as detected by a genetically encoded calcium indicator (NG2-GCaMP6) or a fluoroprobe (Fluo-4). Knocking down α-SMA expression with RNA interference or fixing F-actin with phalloidin or calcium antagonist amlodipine prevented constrictions, suggesting that constrictions resulted from calcium- and α-SMA-mediated pericyte contractions. Carbenoxolone or a Cx43-selective peptide blocker also reduced calcium rise, consistent with involvement of gap junction-mediated mechanisms in addition to voltage-gated calcium channels. Pericyte calcium increase and capillary constrictions became significant after 1 h of ischemia and were coincident with depletion of peri-microvascular glycogen, suggesting that glucose derived from glycogen granules could support pericyte metabolism and delay ischemia-induced microvascular dysfunction. Indeed, capillary constrictions emerged earlier when glycogen breakdown was pharmacologically inhibited. Constrictions persisted despite recanalization but were reversible with pericyte-relaxant adenosine administered during recanalization. Our study demonstrates that retinal ischemia, a common cause of blindness, induces α-SMA- and calcium-mediated persistent pericyte contraction, which can be delayed by glucose driven from peri-microvascular glycogen. These findings clarify the contractile nature of capillary pericytes and identify a novel metabolic collaboration between peri-microvascular end-feet and pericytes.

**Electronic supplementary material:**

The online version of this article (10.1186/s40478-019-0761-z) contains supplementary material, which is available to authorized users.

## Introduction

When Rouget discovered pericytes in 1873, he also suggested that these cells might have contractile capacity and regulate microcirculatory blood flow because of their position on microvessels [1]. After many years of debate, it is now generally agreed that pericytes along the microcirculation express the contractile protein α-SMA (as first shown by Herman and D’amore, 1985) [2] to varying degrees and, contract with physiological and pharmacological stimuli as well as under pathological conditions [3–11]. Pericytes with contractile capacity have been identified in several microvascular beds in addition to the retina and brain, where their role in maintaining blood retina/brain barrier and regulating neurovascular coupling at the microcirculatory level have been well-documented [12]. Similarly, cochlear pericytes also express α-SMA [13] and are thought to be involved in regulation of cochlear microcirculation [14, 15]. Renal pericytes as well control medullary blood flow by Ca^2+^-depended contractions [16]. Cardiac pericytes, which express α-SMA and possess circumferential processes wrapping capillaries have recently been shown to contract and reduce the capillary diameter after coronary ischemia [17].

Increasing evidence indicates that pericytes are vulnerable cells rapidly responding to injury, thus playing pathophysiological roles in ischemia, Alzheimer’s disease, diabetic retinopathy and spreading depolarization [6, 8, 18–25]. For example, microvessel pericytes contract during cerebral ischemia in vivo and do not relax after re-opening of the occluded artery (i.e. recanalization) leading to an incomplete reperfusion that unfavorably affects tissue recovery [6, 8, 26]. This mechanism has been proposed as one of the causes of the low success rate of recanalization therapies after stroke [27]. Similar to cerebral stroke, retinal ischemia is a serious medical problem occurring in 1 out of 100 people over 60 and may lead to severe vision loss [28]. Based on our recent finding that retinal capillary pericytes are endowed with α-SMA [3], we hypothesized that α-SMA may mediate pericyte contraction during ischemia, which might limit reperfusion after retinal ischemia as in cerebral or cardiac ischemia [6, 8, 17]. To address this, we investigated pericyte contractility in response to ischemia-reperfusion injury both in vivo, using adaptive optics scanning light ophthalmoscopy, and ex vivo. We found that contraction of microvascular pericytes persisted despite reopening of the occluded retinal artery. Furthermore, our data show that focal microvascular constrictions at pericyte locations could be prevented by in vivo fixation of F-actin or silencing α-SMA gene expression by small RNA interference, strongly suggesting that they resulted from α-SMA-mediated contractions.

The cellular mechanisms underlying ischemia-induced pericyte contraction are unclear. Although a calcium and α-SMA-mediated contraction is a likely mechanism as in smooth muscle cells, why the contraction is delayed for at least an hour under in vivo conditions is poorly understood [8, 29, 30]. One hypothesis is that the intracellular calcium may increase in pericytes due to the depletion of energy storages during ischemia. The principal glial cells in the retina, the Müller cells, synthesize, store, and degrade glycogen in their end-feet, which wrap around capillaries and are in direct contact with pericytes [31]. Müller glia are known to rely primarily on aerobic glycolysis to generate ATP and each glycogen granule contains around 30,000 glucosyl units, serving as a reservoir of glucose that can be rapidly mobilized when needed [31]. Thus, microvascular glycogen might provide glucose to maintain pericyte metabolism in the absence of sufficient glucose transported from blood. Here, by using transgenic mice expressing NG2-driven GCaMP6 solely in pericytes (NG2-GCaMP6 mice) or loading pericytes in vivo with Fluo-4, we found that intracellular calcium started rising 40 min after occlusion of the central retinal artery in capillary pericytes. Intravitreal injection of various pharmacological inhibitors showed that calcium increase in pericytes had multiple sources and involved not only voltage-gated calcium channels but also gap junctions. Finally, pericyte calcium increase and capillary constrictions were coincident with depletion of peri-microvascular glycogen.

## Materials and methods

### Animals

One hundred and forty two albino (Swiss or CD1), twenty seven NG2-DsRed [32], and sixteen NG2:GCaMP6 [4] (20–35 g) mice were housed under diurnal lighting conditions (12-h darkness and 12-h light) and fed ad libitum. The NG2:GCaMP6 strain was generated by crossing NG2Cre mice (008533, Jackson Laboratory, Bar Harbor, ME) with floxed GCaMP6 mice (024106, Jackson Laboratory). The number of animals used in each experiment is indicated in the corresponding legend and the Additional file 12: Table S1.

### Study approval

Animal housing, care, and application of experimental procedures were all carried out in accordance with the institutional guidelines and approved by the Hacettepe University Animal Experiments Local Ethics Committee (2012/63), committee guidelines on animal resources at the University of Rochester (Rochester, New York), and the guidelines of the Canadian Council on Animal Care and the Centre de Recherche du Centre Hospitalier de l’Université de Montréal (CRCHUM, Montreal, Quebec, Canada).

### Central retinal artery occlusion and reperfusion

Mice were anesthetized with 50 mg/kg ketamine (i.p., Ketalar, Pfizer, Cambridge, UK) and 10 mg/kg xylazine (i.p., Alfazyne 2%, Alfasan International, Woerden, The Netherlands). Body temperature was monitored by a rectal probe and maintained at 37 ± 0.1 °C using a homoeothermic blanket control unit (Harvard Apparatus, Holliston, MA). Surgery was performed under a surgical microscope (SMZ1000, Nikon Instruments Inc., Amsterdam, The Netherlands) at 4x to 25x magnification. We exposed the optic nerve head and central retinal artery at the level of the orbit and placed a small strip (0.3 × 1 mm) of 20% FeCl_3_–saturated filter paper over the optic nerve for 3 min to interrupt blood flow as previously reported by Karatas et al. for the middle cerebral artery [33]. The FeCl_3_ application leads to formation of a clot that blocks blood flow to the retina. The contralateral eyes served as control. Sixty minutes after topical FeCl3 application, tPA was infused (Actilyse, INN alteplase, Boehringer Ingelheim, Germany) using a syringe infusion pump (NE-1000, New Era Pump Systems, Farmingdale, NY) through a tail vein catheter. Approximately 10% of the typical tPA dose used in mice (10 mg/kg) [33] was injected as a bolus during the first min (50 μl/min) and the rest as a continuous infusion over a 30–60 min interval (15 μl/min). A 60-min time point after ischemia was chosen to allow enough time for pericyte contraction based on previous observations in the brain [8] and to increase the chance of clot lysis. Approximately, 50% of the ischemic animals showed retinal recanalisation starting 30–60 min after tPA injection in accordance with the 50% opening rate of the brain clots using tPA [33]. For experiments involving adaptive optics scanning light ophthalmoscopy (AOSLO) with recanalisation, NG2:GCaMP6 mice, and lectin morphological analysis, ischemia was induced by ligating a 10–0 nylon suture around the central retinal artery to interrupt blood flow through the ophthalmic vessels [34] and the retinal blood flow was restored by releasing the suture.

### Laser speckle Flowmetry

Laser speckle contrast (LSC) imaging was used to monitor blood flow by measuring the velocity of moving red blood cells as previously described [35]. Briefly, mice were placed in prone position and the pupils were dilated with 1% tropicamide (Bilim İlaç, Istanbul,Turkey). A CCD camera (Basler 602F, Basler Vision Technologies, Ahrensburg, Germany) was positioned above the eye (12 cm), and a low power laser diode (785 nm, < 10 mW) was used to diffusely illuminate the retina by placing a mouse contact lens (Ocular Instruments, Bellevue, WA) over the cornea after applying lubricant eye gel (Allergan, Irvine, CA) to visualize the fundus. The field of imaging was adjusted using a variable magnification objective on the microscope (SMZ1000, Nikon Instruments Inc.). Ten raw speckle images were acquired at 100 Hz at intervals of every 10 s, processed by computing the speckle contrast using a boxcar average of 7 × 7 pixels and, averaged to improve the signal-to-noise ratio. Pseudocolored relative blood flow (RBF) images were calculated by computing the relative flows at pre- and post-ischemia conditions. Data is reported relative to a zero flow baseline taken as the RBF obtained from an animal sacrificed at the end of the experiment by anesthetic overdose. To measure the changes in RBF, the regions of interest were placed over 3 non-overlapping randomly selected tissue areas away from large retinal vessels corresponding to microvascular regions.

### In vivo fluorescence retinal angiography

To visualize retinal vasculature by in vivo fluorescence microscopy and monitor the success of the central retinal artery occlusion, a fluorescent dye (FITC–dextran-70S or/and TRITC–dextran-500S) (0.5 mg in 0.1 ml saline, Sigma, St. Louis, MO) was injected through a tail vein catheter. Sixty minutes after tPA injection, the fluorescence angiogram was repeated to confirm whether or not recanalisation of the retina was successful. To assess the microvascular recanalisation, the fluorescence intensity was measured within four regions of interest away from macrovessels (i.e. microvascular area) using in vivo retinal angiography images.

### Ex vivo fluorescence imaging of vasculature on whole-mount retinas

After in vivo recordings, mice were sacrificed by cervical dislocation under anesthesia and the eyes were collected and fixed for 1 h in 4% paraformaldehyde (PFA) at room temperature. Retinas were dissected and flattened as whole-mounts by making four radial cuts, mounted on slides with 1X phosphate buffer solution (PBS) as described [36], and visualized under a fluorescence microscope.

### Retinal immunohistochemistry

Eyes were collected, fixed for 1 h in 4% PFA at room temperature, and the retinas prepared as flattened whole-mounts by making four radial cuts [36]. Whole retinas were labeled with lectin (20 μg/ml in PBS containing 0.5% Triton X-100 (PBST); Vector Laboratories, Burlingame, CA) or antibodies against chondroitin sulfate proteoglycan-4, neural glial antigen-2 (NG2) (Merck Millipore, Billerica, MA), claudin-5 (Life Technologies, Carlsbad, CA), platelet-derived growth factor receptor beta (PDGFRβ) (Abcam, Cambridge, UK), connexin 43 (Cx43) (Sigma), and cellular retinaldehyde binding protein (CRALBP) (Thermo Fisher Scientific, Waltham, MA). Secondary antibodies were: anti-rabbit IgG conjugated to Cy2 (Jackson ImmunoResearch, West Grove, PA), anti-mouse IgG conjugated to Cy3 (Jackson ImmunoResearch), or anti-mouse IgG conjugated to 350 nm fluorophore (Molecular Probes, Invitrogen, Eugene, OR). Briefly, retinas were permeabilized by freezing and thawing in PBST (− 80 °C for 15 min, room temperature for 15 min), washed 3 times for 10 min, and incubated in 2% PBST at 4 °C overnight. The retinas were washed in PBST 3 times for 5 min, incubated in blocking solution (10% fetal bovine serum in PBST) for 1 h at room temperature, and them in each primary antibody in blocking solution (5 μg/ml) at 4 °C overnight. The following day, samples were washed in PBST 3 times for 5 min and incubated in secondary antibody diluted with blocking solution (3 μg/ml) for 4 h at room temperature. We mounted retinas vitreal side up on slides and covered them with anti-fade reagent containing Hoechst-33,258 to label cellular nuclei (Molecular Probes). Retinas were imaged under a light microscope (400x, Eclipse E600, Nikon Instruments Inc.) equipped with a manually controlled specimen stage for X, Y, and Z-axis, a color camera (model DXM1200, Nikon Instruments Inc.), a fluorescent light source (HB-10104AF, Nikon Instruments Inc.), and an image analysis software (NIS-Elements, Version 3.22, Nikon Instruments Inc.). Confocal images of the stained sections were obtained with a Zeiss LSM-510 confocal laser-scanning microscope equipped with a diode laser 488 nm and 561 nm source for fluorescence illumination, and a Leica TCS SP8 DLS (Leica, Wetzlar, Germany) confocal laser-scanning microscope, with a X-, Y-, and Z-movement controller, and a high-resolution PMT (Zeiss, Oberkochen, Germany) and HyD (Leica). We generated panoramic pictures of retina by tiling individual images (20x).

### Ex vivo stereological analysis

We quantified the number of microvessel constrictions, defined as a focal narrowing to < 1/2 diameter of the upstream and/or downstream vessel segment, analyzed whether these constrictions colocalized with pericytes (< 10 μm away from the pericyte soma). For this purpose, antibodies against NG2, claudin-5, and Hoechst 33258 were used to label pericytes, vessels, and nuclei, respectively, followed by a stereological analysis that randomly scanned selected 3D-disector probes across the entire retina as described [37]. We analyzed an average of 137 disectors per retina (same area between animal cohorts), and focused on microvessels with a diameter < 9 μm (capillaries with a diameter below 7 μm encompass > 90% of all capillaries), in the 3D-disector frame (field of view: 400 × 300 μm along Z-axis). First, we quantified the total number of constrictions using claudin-5 or lectin (green channel), followed by the analysis of microvessel constrictions in NG2-positive pericytes (red channel) using merged images. Total microvessel constrictions and colocalization with NG2-positive pericytes were calculated using the fractionator equation as follows: total number of elements = Σ quantified elements / ssf X asf X tsf, where ssf is the section-sampling fraction (ssf = number of sections sampled / total sections = 1), asf is the area-sampling fraction (asf = [a (frame)] / area x-y step between disectors = 0.84), and tsf is the thick-sampling fraction (tsf = frame height / section thickness = 1) [37]. For analysis of vessel diameter, we randomly scanned selected 3D-circular probes across the entire retina (400x, 60 μm radius) and analyzed microvessels (diameter < 9 μm, > 90% of capillaries) and macrovessels (diameter > 9 μm). Then, claudin-5 staining (green channel) was used to measure the outer vascular diameter at any point where a vessel touched the probe. The mean diameter for each animal was calculated from all measurements.

For quantification of the calcium influx, we analyzed Fluo-4-loaded and NG2:GCaMP6 retinas using the disector technique described above. At the end of the experiments, we removed the eyes and fixed for 1 h with 4% PFA and, immediately after, we randomly took pictures of pericytes across the entire retinal whole-mounts by a 40x objective and same exposure time and gain for all cohorts. We quantified well-defined calcium-loaded cells eccentrically placed over the microvessel wall of experimental and control retinas. For quantification of the number of pericytes with a marked increase in intracellular calcium levels we quantified pericytes over a threshold, which was strictly the same for all cohorts (80–150 in 8-bit images for Fluo-4-loaded retinas, control mean ± 2*SEM for NG2:GCaMP6 retinas). The green fluorescence intensity in pericytes was measured manually in ImageJ (National Institute of Health). Background signal of each pericyte was obtained by averaging 3 ROIs placed in the parenchyma, around pericytes. The fluorescence signal in each pericyte was extracted and parenchymal signals subtracted.

For analysis of connexin 43 expression between pericytes and Müller cells, we obtained z-stacks (0.15 μm per slice) of the immunolabeled tissue with apotome fluorescent microscope (Apotome 2, Zeiss) that allowed optical sectioning followed by reconstruction using Imaris software (Bitplane, Zurich, Switzerland) to render a detailed 3D image of signal colocalization. For quantification of glycogen at the surface of microvessels, sagittal sections of the entire eye were obtained (20 μm-thick) and labeled for glycogen using periodic acid-Schiff (PAS) staining [38], and vessels were visualized with lectin. Each retinal section was analyzed using the disector technique, taking 10 disectors per section (400 × 300 μm) for a total of ~ 115 disectors per eye. A semiautomatic computer routine was used to first identify microvessels and any constriction in the fluorescent channel, followed by quantification of glycogen levels (brightness) over the selected microvessels in the bright field channel. Lastly, the fractionator technique was applied to experimental and control groups, as we described above.

### Short interfering RNA (siRNA) in vivo knockdown

A custom-designed, in vivo specific HPLC purified α-SMA siRNAs and a scrambled silencer select negative control siRNA were purchased (4,457,308 and 4,404,020, respectively, Ambion LifeTech, Carlsbad, CA). This siRNA was previously studied in wound healing experiments in the murine liver [39]. Each siRNA was injected into the vitreous chamber using a 34-gauge Hamilton syringe (0.5 mg/ml siRNAs, total volume: 3 μl). Prior to injection, siRNAs were mixed with a transfection reagent to facilitate cell entry in vivo. Briefly, a transfection mixture composed of 3 μl In vivo-jetPEI (PolyPlus transfection, 201-10G, Illkirch-Graffenstaden, France) and 12.5 μl of 10% Glucose in 9.5 μl of nuclease free water was added to the nucleic acid mixture (3.76 μl from 25 μg siRNA, 12.5 μl of 10% Glucose in 8.74 μl of nuclease free water), and incubated for 15 min at room temperature. Transfection mixture was prepared fresh before each knockdown experiment. Forty-eight hours after intraocular siRNA delivery, mice were sacrificed. In all knockdown experiments one retina of the animal was used for α-SMA siRNA delivery and the contralateral retina was used as control siRNA delivery.

### Adaptive optics scanning light ophthalmoscopy (AOSLO)

AOSLO was used to simultaneously image capillary blood flow and pericytes in vivo as previously described [32, 40, 41]. For AOSLO imaging, mice were anesthetized with 100 mg/kg ketamine and 10 mg/kg xylazine (i.p.), and positioned in a stereotaxic stage that allowed adjustments in rotation, azimuth, and angle as well as x, y, and z translation for precise alignment with the AOSLO imaging beam. A rigid contact lens (0–10 diopter 1.55–1.6 mm base curve) was placed on the cornea to maintain hydration and optical quality (Unicon Corp., Osaka, Japan), and 1% topical tropicamide and 2.5% phenylephrine (Alcon, Fort Worth, TX) were applied to dilate the pupil and freeze accommodation. Animals were maintained on 0.5–1.5% isoflurane anesthesia throughout the imaging, and a heat pad was used to maintain normal body temperature. AOSLO Imaging comprised three coaxial light channels, a visible 514 nm channel (Argon CVI Melles Griot, Albuquerque, NM), a near-infrared (NIR) imaging source (796 nm low-coherence superluminescent diode, Superlum, Carrigtwohill, Ireland), and 830 nm NIR wavefront sensing source (Qphotonics, Ann Arbor, MI). Power levels were: 200–220 μW for 796, 20–40 μW for 830 nm and 100–460 μW for 514 nm measured at the cornea. Light was raster scanned by two mirrors operating at 15.45 kHz (resonance scanner) and 25 Hz (galvanometric scanner). Optical aberrations were measured at the pupil by a Hartmann-Shack sensor composed of a 7.8-mm focal length lenslet array (Adaptive Optics Associates, Cambridge, MA) focused on a Rolera XR camera (QImaging, Surrey, BC). Wavefront correction was provided by a deformable mirror (DM97 ALPAO, Biviers, France) operated in closed-loop mode at 13 Hz. Back-scattered NIR light and visible fluorescence were detected by two Hamamatsu photomultiplier tubes (PMTs) optimized for visible and NIR spectra (H7422–40 and − 50; Hamamatsu, Shizuoka-Ken, Japan). DsRed fluorescence was detected using a 2 × 630/92 bandpass filters (Semrock Inc. Rochester, NY). A pinhole was placed in front of the PMT detectors to provide confocal imaging (50 μm, 514-nm channel; 25 μm, 796-nm channel). Combined, the three channels provide wavefront sensing, blood flow imaging and DsRed pericyte imaging in simultaneous acquisition at a frame rate of 25 Hz capture. Fluorescence frames (50 to 3000) were averaged to increase signal to noise ratio of the dim fluorescence evoked at low power levels used to prevent photo damage of the retina, which is maximally stimulated in the visible regime. Video data was spatially registered on a frame-by-frame basis to remove artifacts of biological origin (eye movement, respiration and heart rate artifacts). Video data was registered by an approach described in detail by Dubra and Harvey [42]. Briefly, a reference frame was manually selected by the user that contained minimal warping and distortion caused by eye motion. All other frames within the video were then registered to the reference frame. Videos were registered in full-frame mode.

### Analysis of AOSLO images

The diameter of pericyte soma in AOSLO images was manually measured perpendicularly to the capillary axis using ImageJ (Image J, National Institute of Health, Bethesda, MD). The pericyte intensity cross section value was analyzed using a software developed for this purpose (R Foundation, Vienna, Austria), which aligns fluorescence intensity patterns for each individual pericyte and compares changes in the fluorescence intensity pattern along the probe and under different experimental or control conditions. To confirm the validity of the fluorescent signal, the pericyte basement membrane was also labeled ex vivo using lectin. The length of the X and Y axes were measured to obtain a pericyte shape factor (*f* = x/2y), which indicated whether the soma is placed along the vessel wall (*f* > 1, horizontal ellipse) or protruded away from the vessel wall (f ≤ 1, sphere/vertical ellipse). Capillary lumen diameter was measured using the same approach by evaluating the motion-contrast provided by the movement of erythrocytes in the NIR channel [32]. This approach provides the inner luminal diameter by computing the temporal variance of pixels in the NIR channel resulting from erythrocyte movement. Because this strategy relies on erythrocyte movement detectable only in capillaries with blood flow, microvessels were also examined ex vivo on whole-mounted retinas and post-hoc at the end of the AOSLO imaging experiments. For both types of analyses, frames were computed, registered and summed to increase signal-to-noise ratios of the fluorescence (DsRed) and reflectance (capillary lumen) channels.

### Intraocular drug delivery

The following agents were intravitreally injected 1–2 h prior to ischemia (volume: 1–3 μl) at ~ 1 mm posterior to the limbus using a glass micropipette (tip diameter = 50–90 μm): i) adenosine (1 mg/ml, Molecular Probes), ii) carbenoxolone (CBX) (30 μM, Sigma), iii) amlodipine (1 mg/ml, Pfizer); iv) propidium iodide (PI) (1 mg/ml, Molecular Probes), v) fluorescent phalloidin (200 U/ml, Biotium, Fremont, CA), non-fluorescent phalloidin (200 U/ml, Merck Millipore), and vi) Fluo-4 (5 μM, Invitrogen, Carlsbad, CA). All agents injected in each experiment are indicated in the Table 1. Animals were sacrificed 1 h after ischemia, with the exception of those treated with PI or Fluo-4, which were sacrificed at 4–6 or 2 h after ischemia, respectively. Intraocular adenosine injection was used to confirm reversibility of pericyte contractions. Intravitreal injection of 1,4-Dideoxy-1,4-imino-D-arabinitol hydrochloride (DAB, 2.5 M, Sigma) was performed with a 34-gauge Hamilton injector. Two hours after DAB delivery, mice were either sacrificed or subjected to retinal ischemia for 30 min after which mice were sacrificed and the retinas collected and prepared for immunohistochemistry.

**Table 1:** Agents administered to the mouse. The table summarizes all agents injected including company, injection site, volume, concentration, vehicle, administration time, sacrifice time, and number of retinas used

Agent	Company	Injection route	Volume	Concentration (Vehicle)	Time of administration	Time of sacrifice	n of mice
Tissue plasminogen activator (tPA)	Actilyse; INN alteplase, Boehringer Ingelheim, Germany	Tail vein	50 μl/min (1 min) + 15 μl/min (30–60 min)	10 mg/kg	1 h after ischemia	30–60 min after tPA injection	17
Dextran (FITC–70S or/and TRITC–500S)	Sigma	Tail vein	0.2 ml	5 mg/ml (saline)	1 h before ischemia and 1 h after tPA injection	1 h after ischemia/recanalisation	34
Adenosine	Molecular Probes	Intravitreal	3 μl	1 mg/ml (water)	Just before ischemia	1 h after ischemia	4
					1 h after ischemia	2 h after ischemia	3
Amlodipine	Pfizer	Intravitreal	3 μl	1 mg/ml (water)	1 h before ischemia	1 h after ischemia	3
Carbenoxolone (CBX)	Sigma	Intravitreal	3 μl	30 μM (water)	Just before ischemia	1 h after ischemia	4
Fluorescent phalloidin	Biotium	Intravitreal	3 μl	200 U/ml (water)	1–2 h before ischemia	1 h after ischemia	5
Propidium iodide (PI)	Molecular Probes, Invitrogen	Intravitreal	1 μl	1 mg/ml (water)	1 h after ischemia	4–6 h after ischemia	5
Tissue plasminogen activator (tPA) + Fluorescent phalloidin	Actilyse; INN alteplase, Boehringer Ingelheim, Germany; Biotium	Tail vein Intravitreal	50 μl/min (1 min) + 15 μl/min (30–60 min) 3 μl	10 mg/kg 200 U/ml (water)	1 h after ischemia 30 min after ischemia	1 h after tPA injection	6
Fluo-4	Molecular Probes, Invitrogen	Intravitreal	3 μl	5 μM (0.005% pluronic acid, 0.00025% cremophor and 0.05% DMSO in artificial CSF)	Just before ischemia	2 h after ischemia	18
Scrambled peptide	Smart-Bioscience	Intravitreal	2 μl	20 μM (nuclease free water)	1 h before ischemia	1 h after ischemia	6
Peptide	Smart-Bioscience	Intravitreal	2 μl	20 μM (nuclease free water)	1 h before ischemia	1 h after ischemia	6
1,4-Dideoxy-1,4-imino-D-arabinitol hydrochloride (DAB)	Sigma-Aldrich	Intravitreal	3 μl	2.5 M (saline)	2 h before ischemia	30 min after ischemia	9
Scrambled siRNA	Ambion® LifeTech,	Intravitreal	3 μl	0.5 mg/ml (10% Glucose, nuclease free wáter, in~vivo-jetPEI®, 10% Glucose)	48 h before ischemia	1 h after ischemia	3
α SMA siRNA	Ambion® LifeTech,	Intravitreal	3 μl	0.5 mg/ml (10% Glucose, nuclease free wáter, in~vivo-jetPEI®, 10% Glucose)	48 h before ischemia	1 h after ischemia	3

### Intravitreal injection of connexin-43 peptide

Connexin-43 (Cx43) mimetic peptide was previously shown to block Cx43 gap junction channels in vivo [43–45]. Cx43 mimetic peptide5 (sequence: VDCFLSRPTEKT) and its scrambled sequence (sequence: TVRPLDSKTCFE) were custom manufactured in TFA-free form by Smart Bioscience (Saint-Egreve, France). Peptides were dissolved in nuclease free water and, 2 μl at 20 μM final concentration was delivered to retina intravitreally before ischemia. Retinal ischemia was performed 1 h later. Just before the ischemia, 2 μl at 5 μM final concentration of Fluo-4 dye was injected to vitreous. Mice were sacrificed 1 h after ischemia and whole-mount retinas were prepared and stained with Texas Red labeled lectin (Vector Laboratories). Peptide 5-treated non-ischemic retinas were additionally used as negative control. Retinas were imaged by confocal microscope.

### Statistical analysis

All values are provided as the mean ± standard error of the mean (S.E.M.). We evaluated all cohorts with normality (Shapiro-Wilk test) and variance (F-test) tests. We compared values of the RBF, number of junctional and helical pericytes, in vivo microvascular diameters, Cx43 expression and density, and total glycogen amount by means of two tailed Student’s *t*-test. For multiple comparisons, we used Analysis of Variance (ANOVA) followed by Dunnett’s or Tukey’s test where appropriate. Moreover, we also analyzed repeated measures data by fitting a mixed effects model. *P* ≤ 0.05 was considered significant. The Pearson product-moment correlation coefficient was used to test potential correlations. All regression lines of frequency distribution graphs were fit with the same order between cohorts.

## Results

### Retinal ischemia induces persistent pericyte contraction

**Fig. 1:**
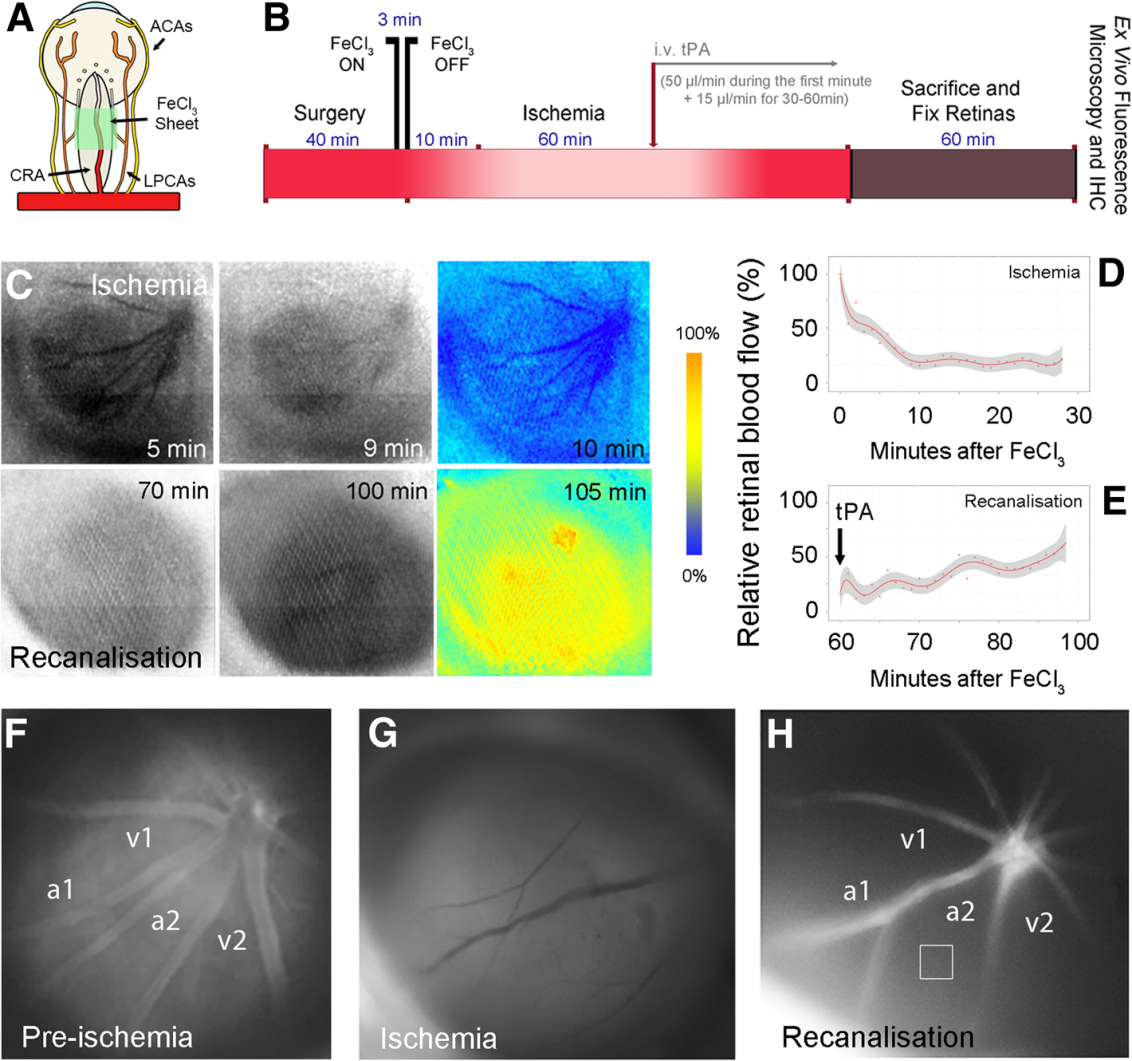
Incomplete retinal microcirculatory reperfusion after ischemia. **a** The illustration depicts the placement of a small strip (0.3 × 1 mm) of 20% FeCl3–saturated filter paper over the optic nerve for 3 min to produce retinal ischemia by occluding the central retinal artery (CRA). ACA: anterior cilliary artery, LPCA: long posterior cilliary artery. **b** The diagram depicts in vivo ischemia/recanalisation experiments and preparation of retinae for ex vivo studies. **c** Raw (black and white) and relative (pseudo-colored) laser speckle contrast (LSC) images recorded within the first 10 min after FeCl_3_ application (upper row) or within 35 min of tPA infusion (lower row) show progress of retinal ischemia and reperfusion after recanalisation. Time (minutes) elapsed after FeCl_3_ placement is indicated on each panel. The relative LSC images at the end of each row were generated by comparing the raw image to the first images in each row to illustrate the relative changes in retinal blood flow. Cold colors (blue) depict a decrease, whereas hot colors (yellow, red) represent an increase in blood flow relative to the beginning of ischemia or tPA infusion. Note the blood flow decrease in vessels as well as retina during ischemia. **d** Illustrates the typical retinal blood flow drop measured in a mouse from 3 non-overlapping randomly selected region of interests over the retina within 10 min after induction of clot formation by FeCl3 in the central retinal artery as detected by LSC imaging. Red dots represent the individual measurements obtained every 10 s, and the red line and shaded area illustrate the mean and its standard deviation, respectively. **e** Illustrates the retinal blood flow recovery within 35 min following the beginning of tPA infusion relative to the maximum flow drop during ischemia. **f**-**h** In vivo fluorescence retinal angiograms obtained with infusion of FITC–dextran-70S (i.v.) before (pre-ischemia) (**f**) or during ischemia (**g**), and after successful recanalisation (**h**). Note less intense fluorescence emission from the retinal tissue (square) and draining venules (v1, v2) after recanalisation despite re-filling of the retinal arterioles (a1, a2), suggesting impaired microcirculatory reflow

**Fig. 2:**
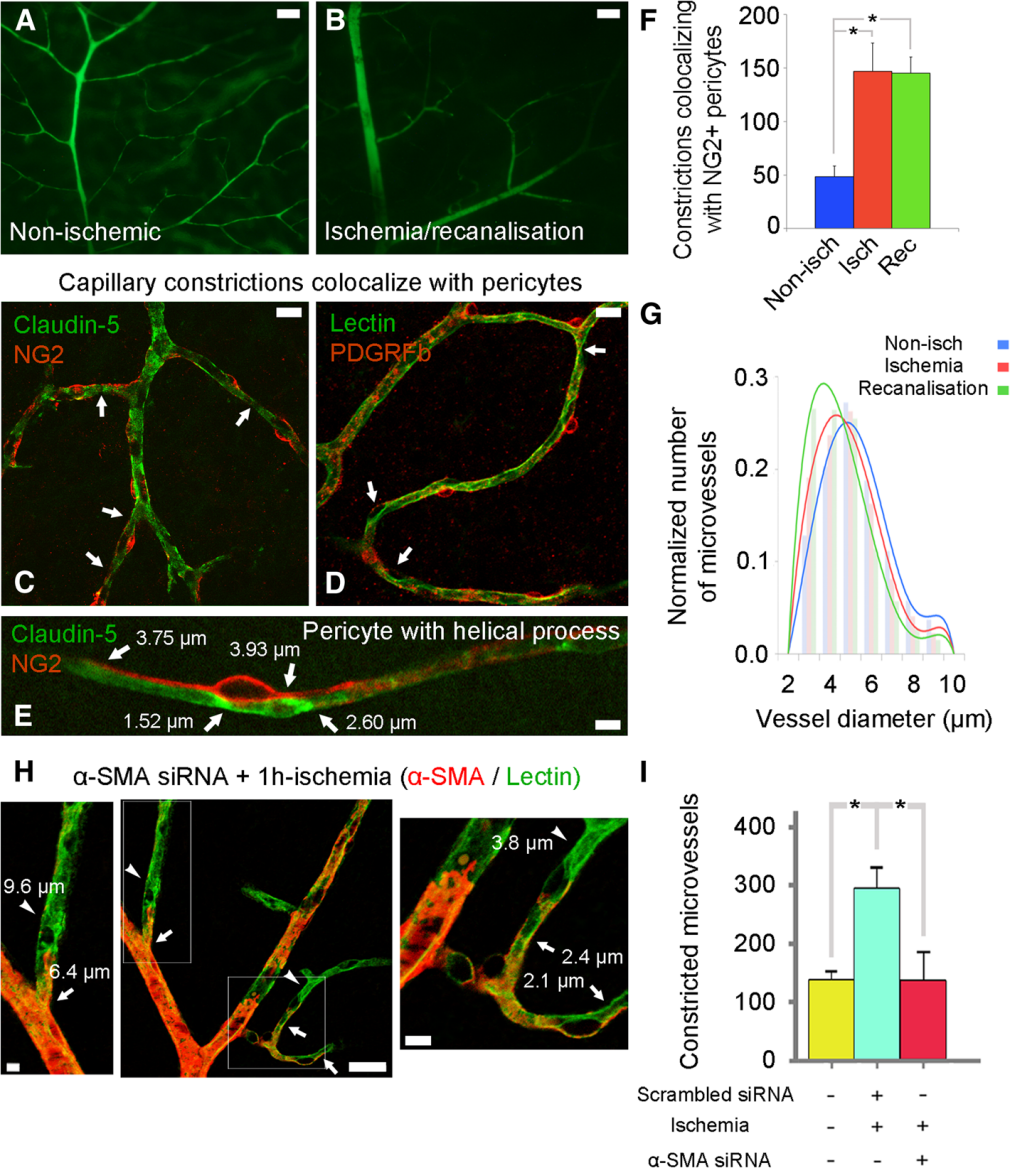
Ischemia-induced microvascular constrictions are caused by pericyte contraction mediated by α-SMA. **a** and **b** Ex vivo fluorescence imaging of vasculature filled with FITC–dextran-70S on whole-mounted retinae from non-ischemic and recanalized eyes confirms incomplete filling of microcirculation after recanalization (**b**) compared to the well-visualized microvessels in a non-ischemic retina (**a**). **c**-**e** Ex vivo co-labeling of the retinae with anti-claudin-5 and anti-NG2 antibodies (**c** and **e**) or lectin and anti-PDGFRβ antibody (**d**) shows that several capillary segments (green) under the pericyte soma or processes (red, in **c** and **d**) were constricted (a focal narrowing to < 1/2 diameter, arrows) in retinae subjected to 1 h of central retinal artery occlusion and 1 h of recanalisation. Note that, in addition to the constricted segments under the junctional pericytes, constrictions were also present near the pericytes with helical processes located at the straight parts of capillaries (**e**, see also the green channel separately in Additional file 1: Figure S1). **f** The number of microvessel (< 9 μm) constrictions (measured within < 10 μm away from or under pericyte soma) in a whole-mount retina that colocalized with NG2+ pericytes increased significantly with ischemia (non-ischemia: *n* = 5 retinae; ischemia: *n* = 3 retinae; *P* = 0.004, ANOVA and Dunnett’s test) and did not recover after recanalisation, (recanalisation: *n* = 5 retinae; *P* = 0.002 compared to non-ischemia, ANOVA and Dunnett’s test). **g** The diameter of the microvessels was also reduced after ischemia (red; *n* = 4268 microvessels in three retinae) and recanalisation (green; *n* = 4533 microvessels in three retinae) compared to the non-ischemic retina (blue; *n* = 5444 microvessels in three retinae). The diameter reduction was more evident in small microvessels. **h** and **i** Knocking down α-SMA expression prevented 1 h-ischemia-induced constrictions. **h** Illustrates microvessels from an ischemic retina pre-treated with α-SMA siRNA 24 h before induction of ischemia. α-SMA was immunostained red and vessels were labeled green with lectin. There were no constrictions at capillary segments where α-SMA expression was knockdown (only green labeled, arrowheads), whereas constrictions continued (arrows) at segments where α-SMA expression was preserved (red). Note that the capillary on the right bottom had a smaller radius at the proximal part where α-SMA was still expressed whereas its distal segment had a larger diameter and no constrictions (inset on the right). A similar pattern is also seen in the left upper capillary (inset on the left). **i** Graph illustrates the number of constrictions in non-ischemic, scrambled siRNA-pretreated ischemic and α-SMA siRNA-pretreated ischemic retinae (*n* = 3 retinae per group; *P = 0.007, ANOVA and Tukey’s test). Scale bar in **a**-**b** = 50 μm; in **c**-**d** = 10 μm; in **e** = 5 μm; in **h** = 5 μm (left), 25 μm (middle), 5 μm (right)

Retinal ischemia was performed by 3-min application of FeCl_3_ over the central retinal artery posterior to the eyeball, which leads to intrarterial thrombus formation by generating oxygen radicals [33] (Fig. 1a, b). Following this procedure, a marked decrease in retinal blood flow was recorded using laser speckle contrast imaging (Fig. 1c, d). Successful retinal ischemia was also confirmed by in vivo FITC-dextran fluorescence and TRITC–dextran-500S angiography (Fig. 1f, g). Following 1 h of ischemia, tissue plasminogen activator (tPA) administration led to restitution of blood flow in retinal vessels within 40 min of infusion in approximately half of the animals; consistent with the 50% recanalization rate of the middle cerebral artery clots by tPA [33] (Fig. 1b, c, e). Refilling of the retinal arterioles was confirmed by fluorescent angiography (Fig. 1h). Nonetheless, the microvasculature could be only partially visualized despite re-filling of the retinal arterioles, indicating compromised microvessel patency (Fig. 1h). The intensity of the retinal tissue fluorescence emission measured in 4 regions of interest (ROIs) away from macrovessels (i.e. microvascular area; square in Fig. 1h) decreased from 115 ± 9 (arbitrary units, a.u.) to 40 ± 2 a.u. during ischemia and did not fully recover after recanalization (51 ± 3 a.u.) compared to pre-ischemic values (*P* < 0.001, ANOVA Tukey’s test). Indeed, ex vivo fluorescence imaging of vasculature filled with FITC–dextran-70S on whole-mounted preparations of recanalized retinas confirmed incomplete filling of the microcirculation (Fig. 2a, b).

To examine whether incomplete filling of the microcirculation correlated with pericyte-mediated capillary constrictions, we colabeled whole-mounted retinas using the pericyte markers NG2 and PDGFRβ with the vasculature markers claudin-5 and lectin. We defined constrictions as a focal narrowing to < 1/2 diameter of the upstream and/or downstream vessel segment and counted them by unbiased stereological methods and analyzed whether these constrictions were colocalized with pericytes (< 10 μm away from each side of the pericyte soma). Our data demonstrate that the constricted microvascular segments corresponded to pericyte locations (Fig. 2c-e; Additional file 1: Figure S1A-C). These pericyte-dependent constrictions did not recover after recanalisation (non-ischemia: 48 ± 10, ischemia: 147 ± 27, recanalization: 145 ± 15 constrictions; *P* = 0.004 and *P* = 0.002, respectively compared to non-ischemia by ANOVA and Dunnett’s test) (Fig. 2f). Images from the deeper retinal plexuses, which are primarily composed of capillaries [46], also demonstrated that capillary pericytes constricted during ischemia (Fig. 2d). Constrictions were common; they were detected near 64 ± 5% of the junctional pericytes located at vessel branching points, and 48 ± 3% of the mid-capillary pericytes located on the straight parts of the capillaries (*P* = 0.006, Student’s t-test) (Fig. 2e). Detailed evaluation of all vascular layers demonstrated that the vast majority of constrictions occurred on microvessels smaller than 7 μm in diameter and remained unchanged after recanalization of the central retinal artery, arguing against the possibility that contractions in high order capillaries was secondary to contractions in upstream microvessels (i.e. capillary collapse) (Fig. 2g). The average microvascular diameter (wall-to-wall width) was measured with claudin-5 or lectin staining at any point where a microvessel randomly touched a 3D-circular probe (see Materials and Methods for further details). Microvascular diameter decreased during ischemia or recanalization compared to non-ischemic values (non-ischemia: 4.77 ± 0.02 μm, 4533 vessels; ischemia: 4.42 ± 0.02 μm, 4268 vessels; recanalization: 4.07 ± 0.02 μm, 5444 vessels; *P* < 0.001, ANOVA Tukey’s test).

### In vivo visualization of ischemia-induced pericyte constrictions with AOSLO

**Fig. 3:**
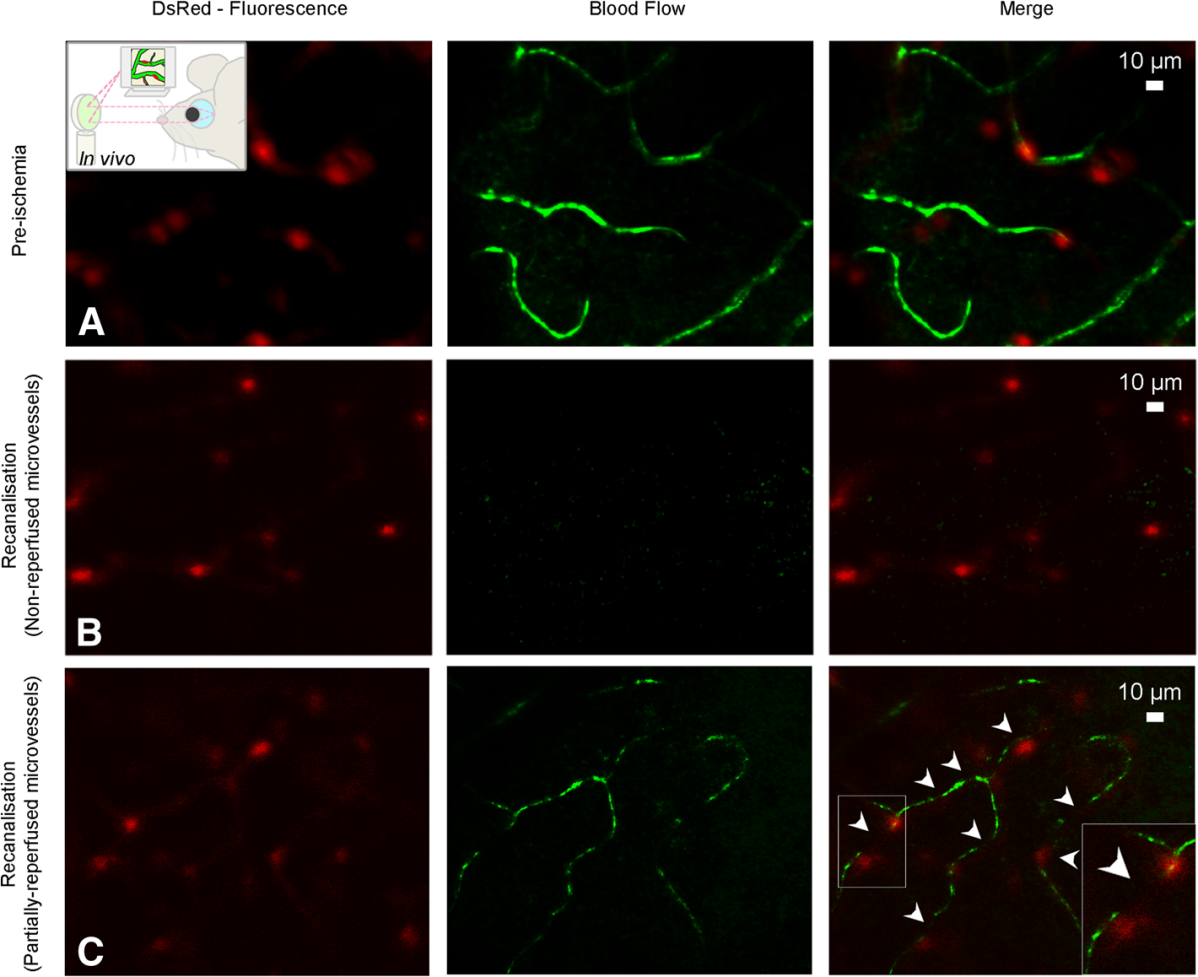
In vivo demonstration of the ischemia-induced pericyte contractions. **a**-**c** AOSLO created an opportunity of observing retinal pericytes on microvessels in NG2-DsRed mice in vivo along with retinal blood flow. **a** Prior to ischemia, pericytes (notably their somas) emitting red fluorescence were visible on microvessels exhibiting an uninterrupted blood flow (green). Vessels were visualized by the motion contrast created by erythrocyte flow and pseudo-colored depending on flow intensity **b** After recanalisation of the occluded central retinal artery, the microcirculation could not be visualized in some microvessels (no-reflow) because AOSLO makes vessels visible by detecting the erythrocyte motion in their lumen (pseudo-colored in green). **c** Some microvessels exhibited a thin stream of flow (incomplete reperfusion). The frequent stalls (black segments, arrowheads) in partially reperfused capillaries is due to reduced perfusion pressure caused by constrictions, some of which correspond to pericyte somas visible in the red channel (inset). Scale bars = 10 μm. For videos, please see the Additional files

**Fig. 4:**
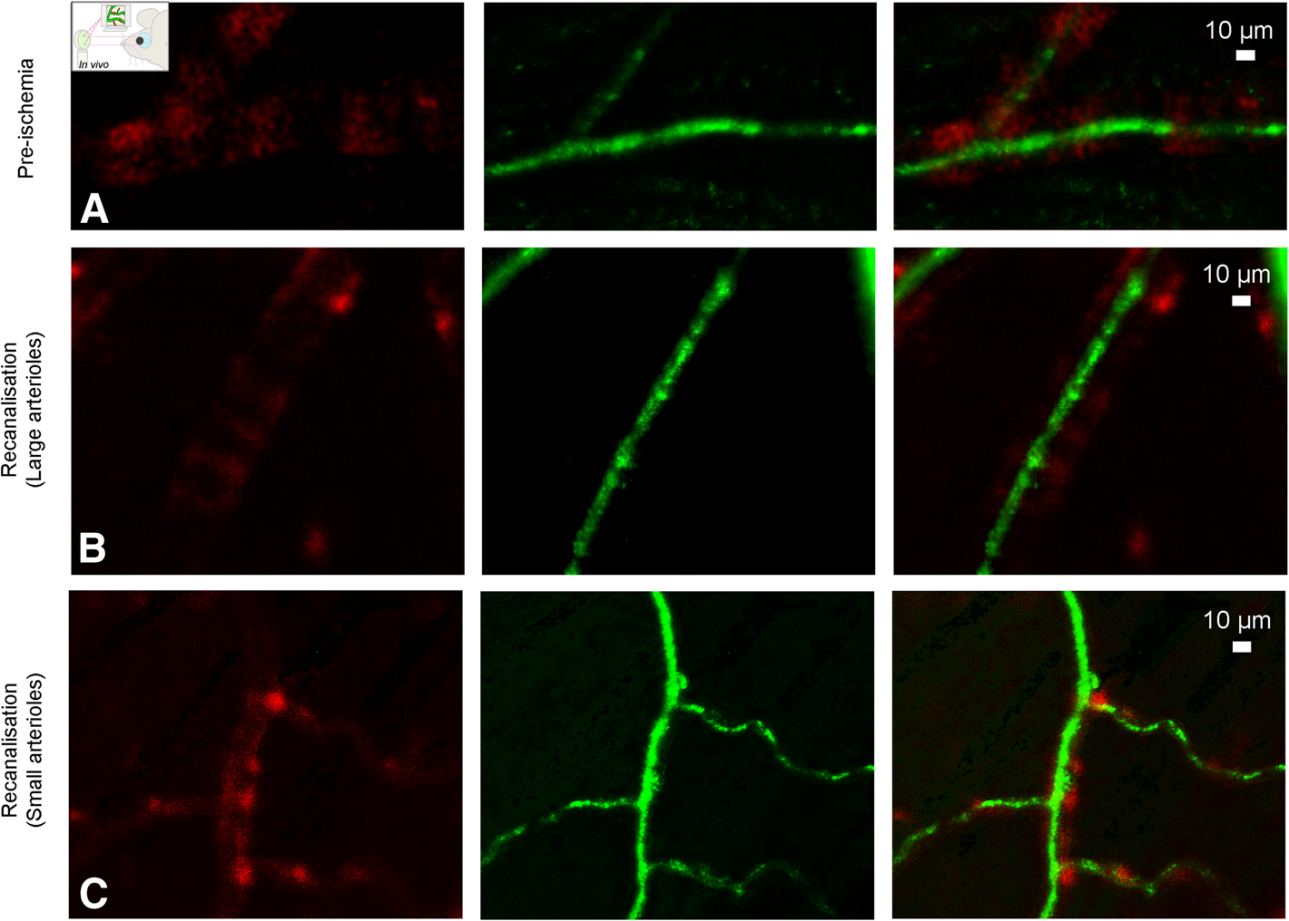
Arterioles were completely reperfused after recanalization and did not exhibit constrictions unlike downstream microvessels. **a**-**c** In vivo AOSLO imaging shows that the blood flow in large (**b**) as well as small arterioles (**c**) was restored to pre-ischemia levels (**a**) after re-opening of the central retinal artery. **b** illustrates one of the arterioles emerging directly from the central retina artery (41.8 μm), whereas the one **c** is a secondary arteriole (22.4 μm). Red fluorescence comes from the smooth muscle cells surrounding arterioles in NG2-DsRed mice, whereas green signal is generated by pseudo-coloring of erythrocyte motion in their lumina. Scale bars = 10 μm. For videos, please see the Additional files

**Fig. 5:**
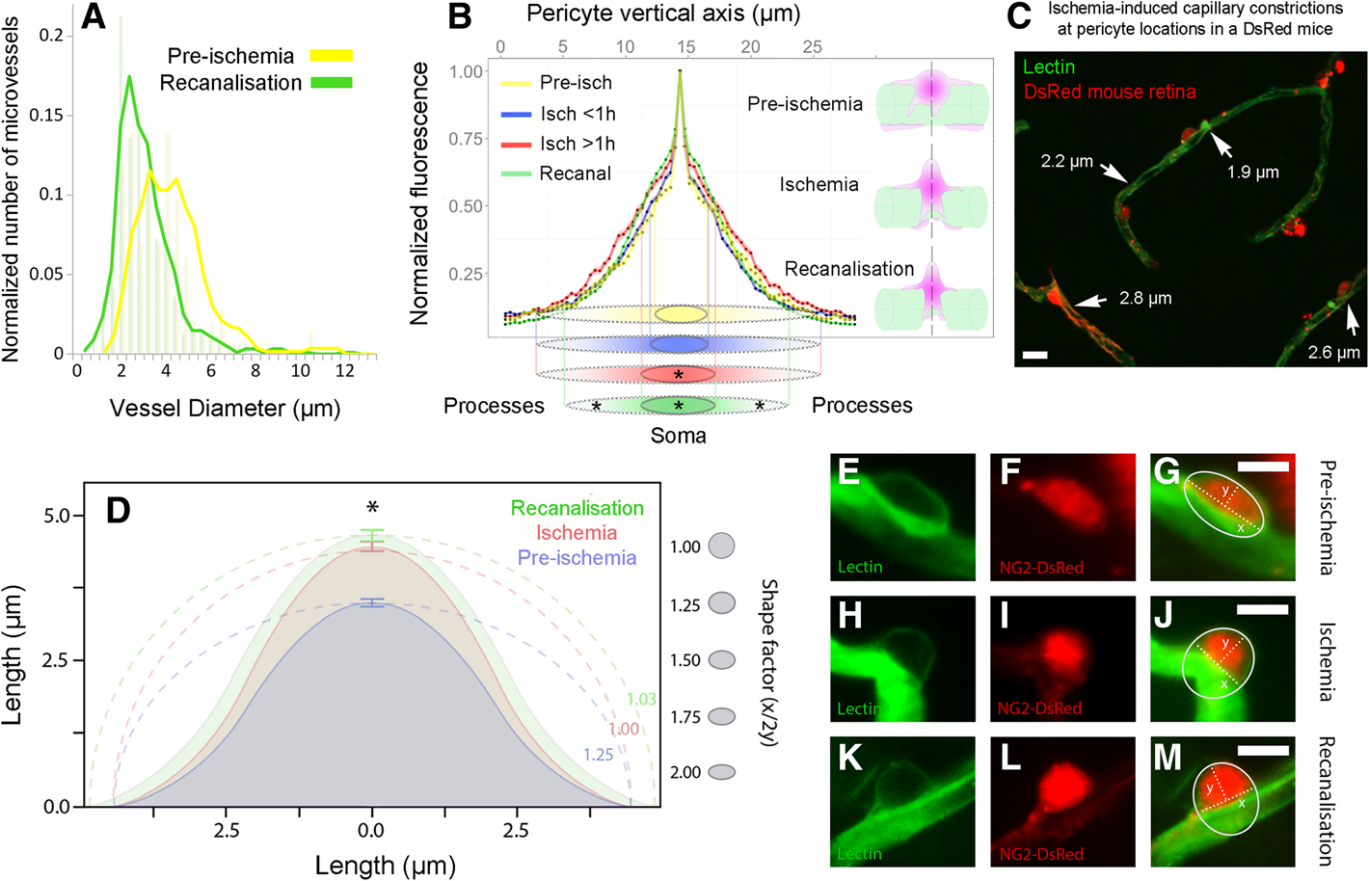
Changes in pericyte morphology during ischemia and recanalisation in vivo suggest pericyte contraction. **a** In vivo luminal diameters of the visualized microvessels (i.e. those with blood flow) at pericyte locations after recanalisation (green; *n* = 441 microvessels in three recanalized retinae) were significantly smaller compared to pre-ischemic retinae (yellow; *n* = 143 microvessels in four pre-ischemic retinae, *P* < 0.0001, Student’s *t*-test). **b** Morphological analysis of DsRed-fluorescent pericytes imaged with AOSLO in vivo disclosed that the pericyte soma rounded up protruding away from the lumen in accordance with a contracted morphology after 1 h of ischemia and remained so after recanalisation. Pericyte shapes at each stage are illustrated on the right and schematically represented at the bottom. The graph shows the distribution of the somatic DsRed signal and, discloses that the soma extended longer along the vertical axis after 1-h of ischemia and during recanalization compared to pre-ischemia (*P* = 0.005, ANOVA and Tukey’s test; yellow, *n* = 72 pericytes in four pre-ischemic retinae; blue, *n* = 226 pericytes in three 0-1 h ischemic retinae; red, *n* = 125 pericytes in four 1-2 h ischemic retinae; green, *n* = 530 pericytes in three recanalized retinae). The vertical lines projected from the 50% of the peak signal values show that the soma fluorescence condensed after 1-h of ischemia and during recanalization in line with a contracted cell body profile (red and green scheme at the bottom). The processes were also shortened along the horizontal axis after recanalisation (green scheme at the bottom, *P* = 0.021, ANOVA and Tukey’s test). **c** Whole-mount retinae from NG2-DsRed mice treated with lectin were examined ex vivo after completing in vivo recordings, which clearly illustrated that the ischemia-induced microvascular constrictions were colocalized with DsRed+ pericytes (arrows, luminal diameters are given next to the arrows). **d** Ex vivo analysis of DsRed+ pericyte morphology after labeling pericyte basement membrane with lectin confirms that soma protruded away from the lumen and assumed a circular shape after 1 h of ischemia (*P* = 0.006, ANOVA and Dunnett’s test) and recanalisation (*P* = 0.01, ANOVA and Dunnett’s test) compared to non-ischemic retinae as illustrated by their shape factor calculated with the formula, *f* = x/2y (x horizontal, y vertical axis and *f* = 1 = sphere, *f* > 1, horizontal ellipse; blue, 1.25 ± 0.06, *n* = 530 pericytes in three non-ischemic retinae; red, 1.00 ± 0.02, *n* = 655 pericytes in three 1 h-ischemic retinae; green, 1.03 ± 0.016, *n* = 387 pericytes in three recanalisated retinae). **e**-**m** Representative examples of pericyte morphology under pre-ischemic (**e-g**), ischemic (**h**-**j**), and recanalisated conditions (**k**-**m**). The basement membrane of NG2-DsRed pericytes (**f**, **i** and **l**) was labeled with lectin (**e**, **h** and **k**). Scale bar in C = 10 μm; scale bar in **e**-**m** = 5 μm


To further establish whether pericytes constrict retinal capillaries during ischemia and recanalization in vivo and to confirm our ex vivo findings, we imaged the retina of NG2-DsRed mice using adaptive optics scanning light ophthalmoscopy (AOSLO). NG2-DsRed mice exhibited phenotypically normal micro and macrovessels (Fig. 3a, 4a, Additional file 5: Movie S1 and Additional file 6: Movie S2), consistent with previous findings [32]. Similar to laser speckle contrast imaging and fluorescence angiography, in vivo AOSLO imaging confirmed the arrest of retinal perfusion following arterial occlusion and incomplete microvessel reperfusion after reopening of the artery. Of particular interest, perfusion was compromised in microvessels (Fig. 3b-c; Additional file 7: Movie S3 and Additional file 8: Movie S4) even after re-opening the central retinal artery (Fig. 4b-c; Additional file 9: Movie S5 and Additional file 10: Movie S6). Reperfused microvessels had smaller than normal diameters at pericyte locations (recanalization: 2.86 ± 0.07 μm, pre-ischemia: 3.53 ± 0.13 μm, *P* < 0.0001, Student’s *t*-test) (Fig. 5a). Of note, the microvessel diameters measured by AOSLO are smaller than ex vivo measurements as AOSLO images the width of the erythrocyte column flowing through but not the wall-to-wall width (compare Fig. 5a to Fig. 2g). Capillary constrictions slowed down or stalled erythrocyte flow mainly at pericyte locations, leading to incomplete microvascular reperfusion in some capillaries (arrowheads in Fig. 3c; Additional file 8: Movie S4) despite normal flow in upstream arterioles (Fig. 4b-c; Additional file 9: Movie S5 and Additional file 10: Movie S6). Morphological analysis of the in vivo pericyte shape (outlined by DsRed fluorescence) along their longitudinal axis (i.e. perpendicular to the capillary axis) showed that the pericyte soma protruded away from the vessel wall starting 1 h after ischemia and remained so after recanalization compared to non-ischemic pericytes (Fig. 5b). The distributions of normalized DsRed fluorescence intensity along the longitudinal axis after ischemia (*P* < 0.0001) and recanalization (*P* = 0.005) were significantly different from pre-ischemia values (ANOVA and Tukey’s test). This observation is consistent with previous reports on the morphology of contracted pericytes both in vivo and in vitro [32, 47, 48]. Our in vivo finding supports the idea that NG2+ microvascular pericytes contract during ischemia, limiting microvessel patency [6, 8]. Since constricted microvessels slowed/arrested erythrocyte flow, the source of motion contrast was largely reduced and some microvessels were no longer visible using this modality (Fig. 3b; Additional file 7: Movie S3) [32]. After completing the in vivo recordings, retinas were examined ex vivo to confirm that visualized capillary constrictions localized at pericyte locations (Fig. 5c). Ex vivo analysis of DsRed+ pericyte morphology by labeling pericyte basement membrane with lectin demonstrated that ischemia/reperfusion caused a substantial increase in soma size along the vertical axis, characteristic of outward protrusion of the contracted pericyte body, relative to the non-ischemic condition (pre-ischemia vs. ischemia, *P* = 0.006; pre-ischemia vs. recanalization, *P* = 0.0204; ANOVA and Dunnett’s test) (Fig. 5d-m). We conclude that ischemia induces capillary constrictions at pericyte locations in vivo, which correlated with quantifiable changes in pericyte soma shape.

**Additional file 5: Movie S1.** Retinal macrocirculation under normal conditions before ischemia in vivo**.** AOSLO movie illustrates 2 large arterioles with blood flow in the retina of a NG2-DsRed mouse under anesthesia. DsRed signal from the smooth muscle cells (SMCs, red channel; sum of 1000 registered frames) is overlapped with the blood flow signal (green channel). (MPG 1180 kb)



**Additional file 6: Movie S2.** Retinal microcirculation under normal conditions before ischemia in vivo. AOSLO movie illustrates the microvessels with blood flow in the retina of a NG2-DsRed mouse under anesthesia. DsRed signal from pericytes (red channel; sum of 1000 registered frames) is overlapped with the blood flow signal (green channel). (MPG 8080 kb)



**Additional file 7: Movie S3.** Retinal microcirculation is not restored after recanalization of the occluded central retinal artery in vivo. AOSLO movie fails to show the non-reperfused microvessels (no-reflow) 10 min after recanalization in a NG2-DsRed mouse under anesthesia. Microvessels could indirectly be identified by tracing the fluorescent pericytes. Pericyte images (red channel; sum of 1000 registered frames) are overlapped with the blood flow (green channel). (MPG 5860 kb)



**Additional file 8: Movie S4.** Some retinal areas present partially restored microcirculation after recanalization of the occluded central retinal artery in vivo. AOSLO movie shows microvessels with weak blood flow 10 min after recanalization in a NG2-DsRed mouse under anesthesia; note interruptions in the blood column (arrowheads) at pericyte locations; microvessels were identified with week flow and by tracing fluorescent pericytes. Pericyte images (red channel; sum of 1000 registered frames) are overlapped with the blood flow (green channel). (MPG 6080 kb)



**Additional file 9: Movie S5.** Retinal arterioles (small) were reperfused after recanalization of the occluded central retinal artery in vivo. AOSLO movie shows the restored blood flow in vessels upstream to microvessels (small arterioles) after recanalization in a NG2-DsRed mouse under anesthesia. Fluorescent retinal smooth muscle cells (SMCs) wrapping arterioles are identified (red channel; sum of 1000 registered frames) with their characteristic ring-shape and overlapped with the blood flow (green channel). (MPG 5330 kb)



**Additional file 10: Movie S6.** Retinal arterioles (large) were reperfused after recanalization of the occluded central retinal artery in vivo**.** AOSLO movie shows the restored blood flow in vessels upstream to microvessels (large arterioles) after recanalization in a NG2-DsRed mouse under anesthesia. Fluorescent retinal smooth muscle cells (SMCs) wrapping arterioles are identified (red channel; sum of 1000 registered frames) with their characteristic ring-shape and overlapped with the blood flow (green channel). (MPG 6040 kb)


### α-SMA is involved in ischemia-induced pericyte contraction

To examine whether the observed ischemia-induced constrictions were caused by α-SMA-mediated pericyte contractions, we pre-treated the retinas with phalloidin, an F-actin stabilizing agent. Phalloidin significantly decreased the number of constrictions compared to the untreated ischemic retinas with or without recanalization (phalloidin pre-treatment + ischemia: 117 ± 21, ischemia: 269 ± 34, recanalization: 305 ± 58 constrictions, *P* < 0.001, ANOVA and Dunnett’s test). Phalloidin treatment also prevented ischemia-induced decrease in microvessel diameter (phalloidin pre-treatment + ischemia: 4.70 ± 0.04 μm, 1993 vessels; non-ischemia: 4.77 ± 0.02 μm, 4533 vessels; *P* = 0.52, ANOVA and Tukey’s test; ischemia: 4.42 ± 0.02 μm, 4268 vessels; *P* < 0.001, ANOVA and Tukey’s test). This finding suggests that F-actin stabilization with phalloidin prevented α-SMA contraction, and hence, luminal narrowing. In line with these findings, knocking down α-SMA expression with RNA interference also significantly suppressed ischemia-induced capillary constrictions (non-ischemic retinae: 139 ± 8 constrictions, scrambled siRNA-treated ischemic retinae: 291 ± 24 constrictions, α-SMA siRNA-treated ischemic retinae: 138 ± 29 constrictions; non-ischemia vs. siRNA+isch, *P* = 0.99, non-ischemia vs. scrambled + isch, *P* = 0.007, siRNA+isch vs. scrambled + isch, *P* = 0.007; ANOVA and Tukey’s test) (Fig. 2h-i; Additional file 1: Figure S1D). As we previously reported [3], α-SMA siRNA had a greater effect on small capillaries (< 7 μm) and prevented ischemia-induced diameter decrease in these capillaries (scrambled siRNA pre-treatment + ischemia: 4.07 ± 0.12 μm, 88 vessels; α-SMA siRNA + ischemia: 4.52 ± 0.12 μm, 92 vessels; *P* = 0.01, Student’s t-test). Together, these results indicate that retinal ischemia induces α-SMA-dependent sustained pericyte contraction leading to capillary constriction. Of note, while our data strongly support that pericytes are responsible for interspaced focal constrictions on microvessels, we cannot entirely exclude some contribution from glial end-feet or endothelial swelling to the longitudinal (non-nodal) microvessel diameter decrease. For example, phalloidin treatment might also have prevented F-actin skeleton reorganization during swelling-induced perivascular end-feet remodeling [49, 50], however, the experiments selectively knocking down of α-SMA expression without affecting other F-actins argue against this possibility.

### Calcium mediates ischemia-induced pericyte contraction

**Fig. 6:**
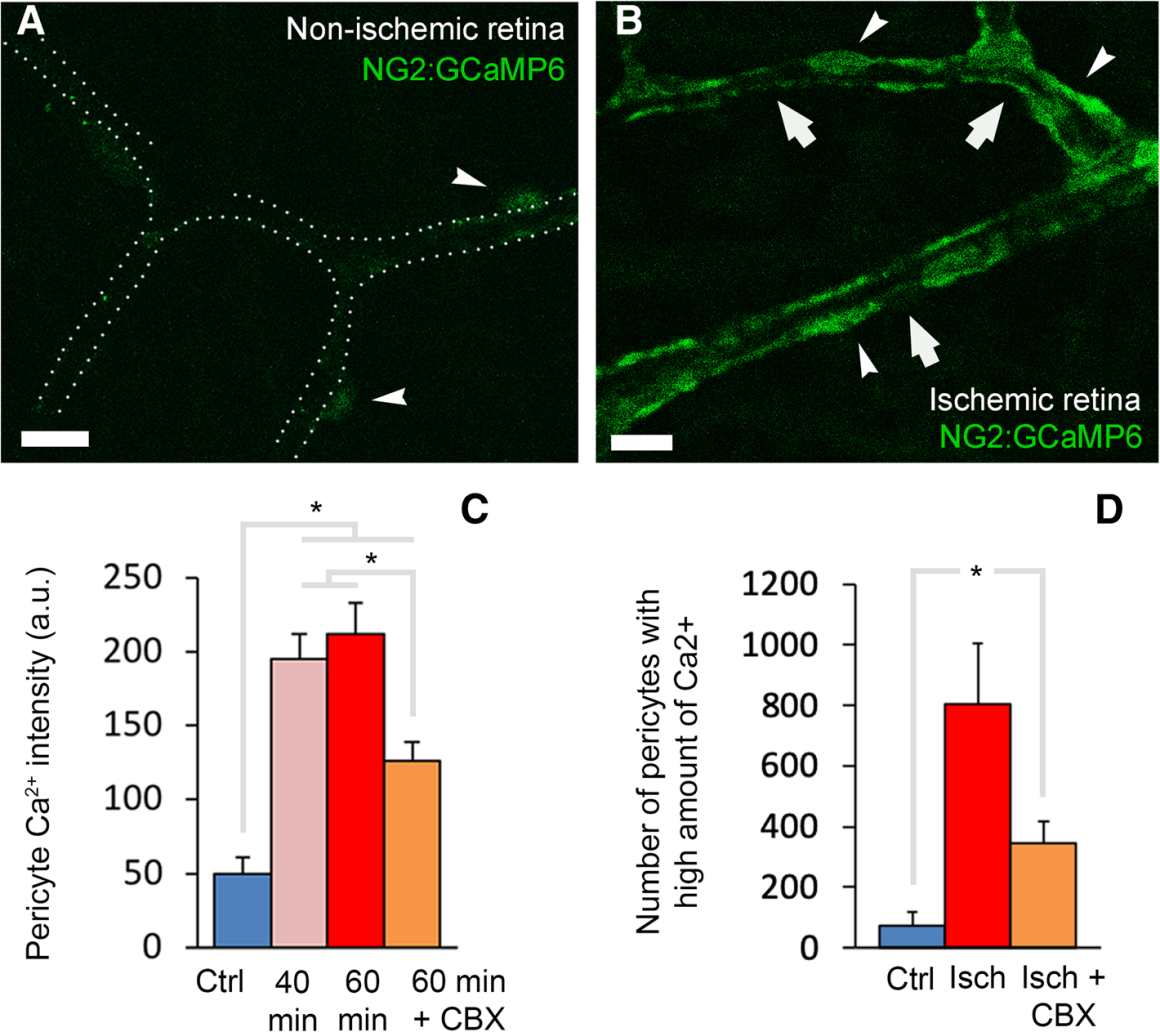
Ischemia-induced pericyte contraction is mediated by calcium and is time dependent in NG2:GCaMP6 mice. **a** and **b** images from the retina of mouse expressing a genetically encoded calcium indicator (GCaMP6) under the NG2 promoter (NG2:GCaMP6) specific for pericytes (arrowheads) and the graphs (**c**-**d**) illustrate that ischemia induces intracellular calcium increase in pericytes (arrows point to constrictions). The intensity of calcium signal increased over time, starting 40 min after arterial occlusion (**c**) (non-ischemic: *n* = 82 pericytes in four retinae; 40-min ischemia: *n* = 91 pericytes in three retinae; 60-min ischemia: *n* = 62 pericytes in three retinae; 60 min ischemia+CBX: *n* = 84 pericytes in five retinae; *P < 0.01, ANOVA and Tukey’s test). The intensity of calcium signal as well as the number of pericytes with high GCaMP6 fluorescence in ischemic retinae was reduced by CBX pre-treatment (**c**-**d**; non-ischemic: *n* = 4 retinae in C and 5 retinae in D; ischemia: *n* = 3 retinae; ischemia+CBX: *n* = 5 retinae; *P < 0.05, ANOVA and Tukey’s test). Most of the intensely calcium signal labeled pericytes were colocalized with microvascular constrictions (arrows in **b**). Scale bars in **a**-**b** = 10 μm

**Fig. 7:**
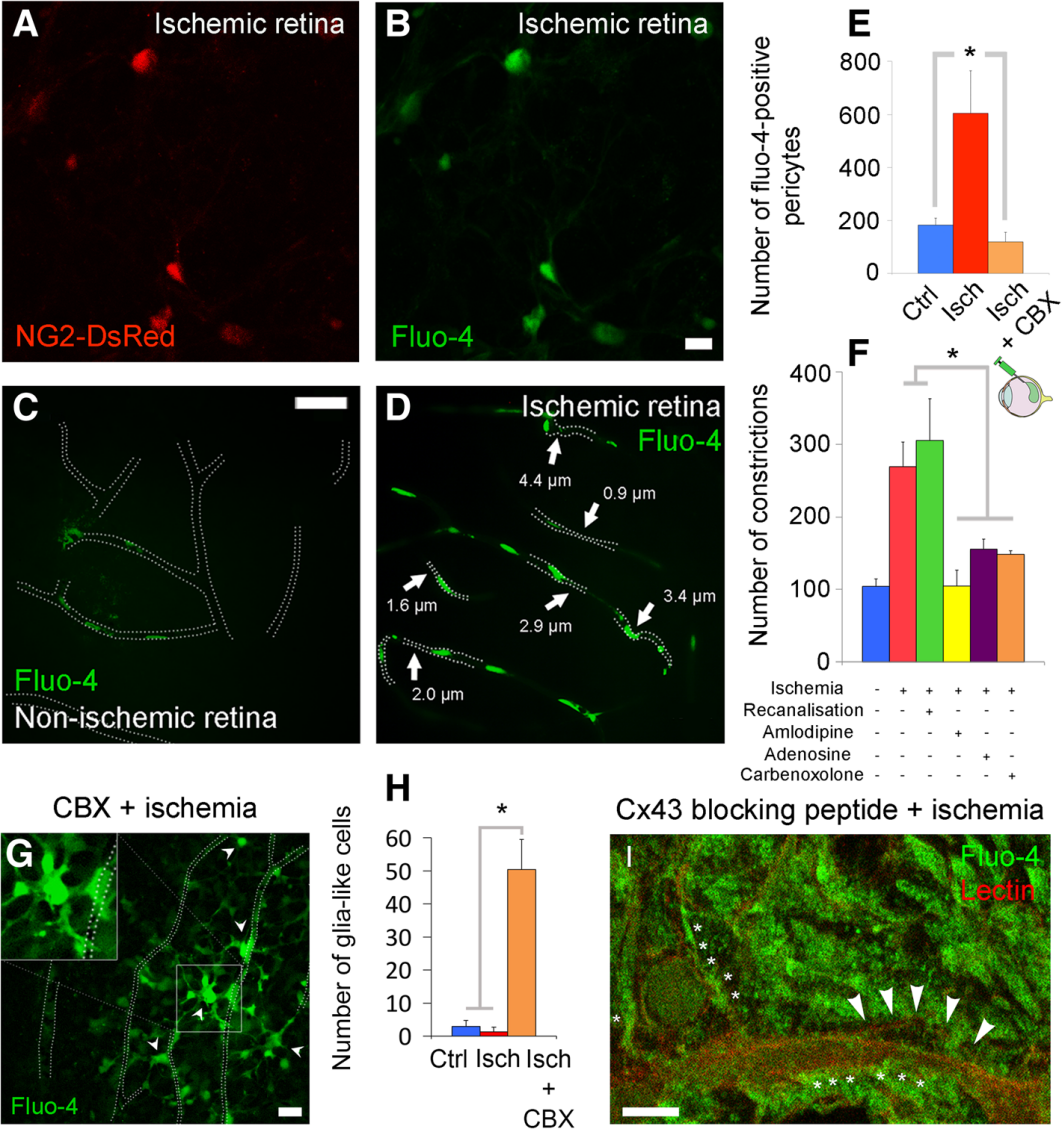
Ischemia induces pericyte contraction by multiple calcium influx pathways. **a**-**f** Intravitreal injection of calcium fluorophore, Fluo-4, to NG2-DsRed (**a**-**b**) or wild type (**c**-**d**) mice labeled only a small number of pericytes in non-ischemic retinae (**c**, **e**), whereas ischemia strikingly enhanced the Fluo-4 signal in pericytes (**b**, **d**-**e**) (non-ischemia: *n* = 7 retinae; ischemia: *n* = 3 retinae; *P* = 0.001, ANOVA and Dunnett’s test). Most of the intensely calcium signal labeled pericytes were colocalized with microvascular constrictions (arrows in **d**). Vessel contours in non-ischemic retinae were traced by following the week Fluo-4 labeling along the vessel wall. Intravitreal administration of gap junction blocker carbenoxolone (CBX) also reduced the number of pericytes labeled with Fluo-4 (**e**; ischemia: *n* = 3 retinae; CBX-treated ischemia: *n* = 4 retinae; *P* = 0.018, ANOVA and Dunnett’s test). **f** Ischemia-induced microvessel constrictions (*n* = 3 ischemic retinae) were prevented by pre-ischemic intravitreal administration of calcium antagonist amlodipine (*n* = 3 retinae; *P* = 0.001, ANOVA and Dunnett’s test), pericyte relaxant adenosine (*n* = 4 retinae; *P* = 0.01, ANOVA and Dunnett’s test), and gap junction blocker carbenoxolone (*n* = 4 retinae), whereas recanalized retinas had similar number of constrictions to the ischemic ones (3 recanalized retinae; *P* = 0.53, ANOVA and Dunnett’s test). **g**-**i** Intravitreal administration of CBX caused glia and their end-feet over the microvessels to become intensely Fluo-4-positive (inset and arrowheads in **g**), suggesting that blockade of gap junctions might have led to calcium rise within glia (**h**; non-ischemia: *n* = 7 retinae; ischemia: *n* = 3 retinae; CBX-treated ischemia: *n* = 4 retinae; *P* < 0.0001, ANOVA and Tukey’s test). **i** The specific connexin-43 blocking peptide also led to a massive increase of intracellular calcium of Müller cell-like structures and their end-feet during ischemia. Arrowheads and asterisks indicate pericyte soma and Muller end-feet surrounding capillaries, respectively. Scale bars in **a**-**b** = 10 μm; in **c**-**d** = 40 μm; in **g** = 20 μm; in **i** = 5 μm

Intracellular calcium increase is thought to mediate pericyte contraction possibly by a mechanism involving α-SMA-myosin interaction as seen in upstream arteriolar smooth muscle cells [2, 51]. Therefore, we next investigated if the ischemia-induced sustained capillary constrictions resulted from unregulated calcium increase in pericytes. For this purpose, we monitored calcium dynamics in NG2:GCaMP6 mice that express a genetically encoded calcium indicator under the NG2 promoter specific for pericytes (Fig. 6a-b). We found that ischemia induced a time-dependent increase in GCaMP6 fluorescence in capillary pericytes starting 40 min after occlusion (Fig. 6c-d). We also injected the calcium indicator Fluo-4 into the vitreous chamber of DsRed transgenic (Fig. 7a-b) or wild type (Fig. 7c-d) mice and examined flat-mounted retinas ex vivo. Intact, non-injured retinas displayed weak Fluo-4 fluorescence in the parenchyma and vascular cells including pericytes (Fig. 7c), identified by eccentric somata location on the microvascular wall as well as by DsRed fluorescence in transgenic mice. In contrast, ischemia led to a marked increase in intracellular calcium levels (Fig. 7b, and d) as evidenced by the large number of pericytes emitting fluorescence over a set threshold (80–150 in 8-bit images) (non-ischemia: 183 ± 26 vs. ischemia: 605 ± 159 Fluo-4-positive pericytes, *P* = 0.001, ANOVA and Dunnett’s test) (Fig. 7e). The parallel results obtained from NG2:GCaMP6 mice rule out the possibility that the increase in calcium signal could result from enhanced Fluo-4 uptake by ischemic pericytes. Consistent with these findings, intraocular injection of amlodipine, a calcium-channel antagonist, prevented ischemia-induced microvascular constrictions (*P* = 0.001, ANOVA and Dunnett’s test) (Fig. 7f, yellow column).

Interestingly, carbenoxolone (CBX), a gap junction blocker [52], significantly reduced the ischemia-induced capillary constrictions (*P* = 0.007, ANOVA and Dunnett’s test) (Fig. 7f, orange column). CBX also decreased the intracellular calcium (*P* = 0.002, ANOVA and Tukey’s test) (Fig. 6c) and the number of pericytes with high intracellular calcium (*P* = 0.034, ANOVA and Tukey’s test) (Fig. 6d) in NG2:GCaMP6 mice or when assessed with fluo-4 (*P* = 0.018, ANOVA Dunnett’s test) (Fig. 7e). While reducing pericyte calcium load, CBX, unexpectedly, led to a calcium increase in surrounding glial cells during ischemia in all animals examined (*P* < 0.001, ANOVA and Tukey’s test) (Fig. 7g-h). To confirm that the above observations with CBX were caused by gap junction blockade, we used a selective connexin-43 (Cx43) inhibitor (peptide-5, INSEC-325). We chose Cx43 because, in the retina, Cx43 is expressed only in glial cells, pericytes and endothelial cells but not in neurons [53]. When we pre-treated retina with peptide-5, we found that it also led to a striking Fluo-4 signal increase in ischemic glial cells and their end-feet as we observed with CBX (Fig. 7i). The scrambled sequence of peptide-5 did not cause a similar increase in glial calcium in the ischemic retina nor did peptide-5 in the non-ischemic retina (Additional file 2: Figure S2A-B). The effect of CBX did not involve inhibition of pannexin-1 channels because intravitreal injection of propidium iodide, a membrane-impermeable fluorescent marker that can pass through open pannexin-1 large-pore channels [54] did not label pericytes, endothelia or glial end-feet (Additional file 3: Figure S3A), whereas it intensely labeled ischemic cells located in the innermost retinal layer (Additional file 3: Figure S3B). This finding indicates that, at this early time point after ischemia, pericytes maintain the integrity of the plasma membrane and their large-pore channels are not open.

**Fig. 8:**
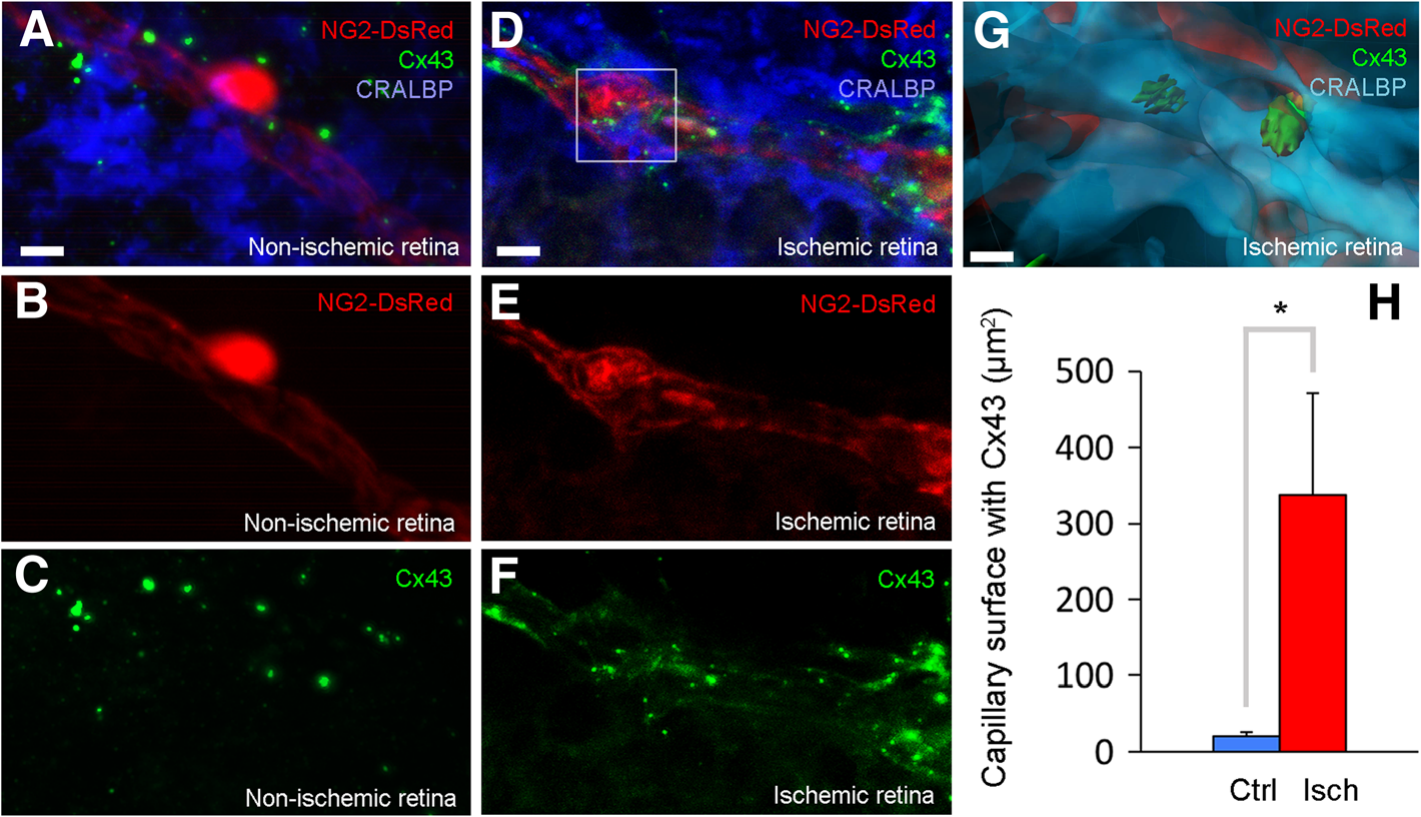
Ischemia induces connexin-43 clustering in Müller end-feet. **a**-**h** Ischemia induced connexin-43 (Cx43, green, cf. **a**, **c** with **d**, **f**), clustering in Müller end-feet (identified with CRALBP, blue, cf. **a** with **d**) over pericytes (NG2-DsRed, red, cf. **a**, **b** with **d**, **e**). **g** 3D reconstruction displays the view of the selected area in **d** by using IMARIS software, and shows Cx43 connexons in Muller end-feet overlying pericytes 60 min after ischemia. All images were captured with optical sectioning followed by a 3D reconstruction. **h** Cx43 expression was much less in non-ischemic retinae and covered a small area on capillary surface in contrast to ischemic retina (**h**; non-ischemia: *n* = 18 pericytes in 3 retinae; ischemia: *n* = 15 pericytes in 4 retinae; *P* = 0.01, Student’s *t-*test). Scale bars in **a**-**f** = 5 μm; in **g** = 2 μm


The observation that gap junction blockage reduces calcium in ischemic pericytes suggests that, albeit indirectly, ischemia may induce calcium signaling (wave) within glia toward their perivascular end-feet, which can release vasoconstrictive mediators through connexin hemichannels opened by ischemia. These mediators can activate their receptors on pericytes and contribute to calcium rise. To test this possibility, we examined the expression of Cx43 and found that ischemia substantially increased Cx43 immunoreactivity over the Müller cell end-feet, identified with the cell-specific marker CRALBP. A 3D analysis to identify Cx43 present in Muller cell end-feet overlying DsRed-positive pericytes (Fig. 8a-g) revealed that CRALBP+ peri-capillary end-feet area exhibiting Cx43 immunopositivity increased from 20.65 ± 5.46 μm^2^ in non-ischemic retina (*n* = 18 capillaries in 3 mice) to 337.32 ± 133.57 μm^2^ in ischemic retina (*n* = 15 capillaries in 4 mice) (*P* = 0.014, Student’s *t*-test) (Fig. 8h). All this quantified perivascular area surrounded DsRed-positive pericytes, suggesting that these connexins clustered in end-feet over the capillaries may contribute to pericyte contractility during ischemia (Fig. 8a-h, Additional file 11: Movie S7).

**Additional file 11: Movie S7.** Ischemia induced connexin-43 expression between pericytes and Müller cells. 3D view of an ischemic retinal section from a NG2-DsRed mouse with pericyte in red (DsRed), Müller cells in blue (anti-CRALBP antibody), and Cx43 in green (anti-Cx43 antibody). 3D view suggests that Cx43 (green) in Müller cell end-feet (blue) may contact with pericyte (red) after 1 h-ischemia. (MPG 2480 kb)


### Ischemia-induced pericyte contraction can be reversed

In vivo retinal ischemia, which induces a milder metabolic challenge owing to collateral blood flow compared to the combination of oxygen-glucose deprivation with iodoacetate and antimycin in vitro [6] did not cause pericyte death within the 1 h-hour period covered by our experiments in line with Neuhaus et al’s findings [55]. Therefore, we tested whether or not the ischemia-induced, calcium-mediated pericyte contraction is reversible shortly after ischemia before irreversible damage occurs [6]. For this, we intravitreally injected adenosine, which relaxes pericytes by hyperpolarizing, hence, decreasing calcium influx [56] before and after ischemia. Adenosine administration 1 h after ischemia significantly reduced the number of pericyte constrictions, as did adenosine given prior to ischemia induction (155 ± 34 and 155 ± 14, respectively vs. 269 ± 34 constrictions in untreated ischemic retinas, *P* = 0.02 and *P* = 0.01, respectively compared to untreated ischemic retinas with ANOVA followed by Dunnett’s test, Fig. 7f, purple column). The average diameter of microvessels randomly measured by the disector technique was also restored (adenosine + ischemia: 4.85 ± 0.03; 3225 capillaries; ischemia: 4.42 ± 0.02 μm, 4268 vessels *P* < 0.001, ANOVA and Tukey’s test). Unlike adenosine, phalloidin post-treatment (administered at reperfusion) was ineffective in relaxing already contracted α-SMA (955 ± 170 constrictions in 3 retinae vs. phalloidin pre-treated ischemic retinas, 117 ± 21 constrictions in 5 retinae). The latter measurement showed the highest number of constrictions because retinae were followed for an additional hour to see the post-treatment effect of phalloidin.

As intravitreally administered amlodipine, adenosine and CBX can also affect large retinal vessels proximal to microvessels, we measured the diameter of upstream vessels in ischemic retinas receiving treatment to test whether the reversal of microvascular constrictions might be secondary to increased flow, hence perfusion pressure, in dilated upstream vessels. No significant difference in luminal size was found between any of the experimental and control cohorts (Additional file 4: Figure S4), indicating that the prevention of microvascular constrictions was independent of upstream vascular changes.

### Perivascular glycogen may delay pericyte contraction

**Fig. 9:**
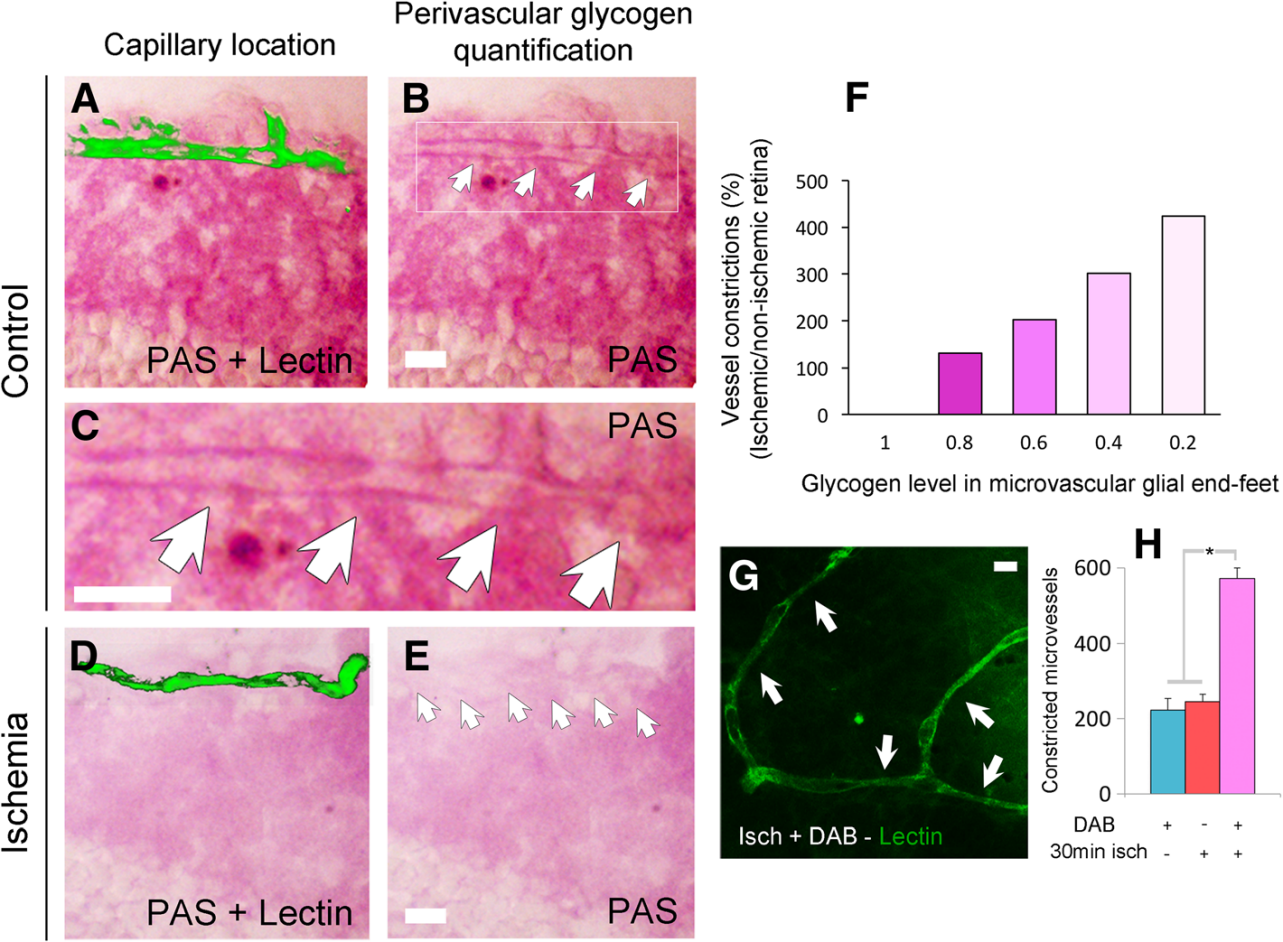
Depletion of glycogen within glial end-feet surrounding microvessels contributes to microvessel constrictions. **a**-**f** When microvessel constrictions emerged 1 hour after ischemia (*n* = 3 retinae), glycogen levels (detected by PAS staining) in microvascular glial end-feet (microvessels were detected by lectin staining, a, d; arrows in b, c, e) were significantly decreased (**d**-**e**) compared to the non-ischemic eye (*n* = 3 retinae) (**a**-**c**). PAS staining was performed after treatment with dimedone to block aldehyde groups on non-glycogen substances. The number of microvessel constrictions was highly correlated with low levels of end-feet glycogen during ischemia (Pearson product-moment correlation, *R*^*2*^ = 0.992; *P* < 0.001) (**f**). **g** and **h** Inhibition of glycogen utilization by DAB exacerbated the ischemia-induced constrictions so that they appeared as early as 30 min after ischemia (arrows, **g**). **h** The number of microvascular constrictions significantly increased 30 min after ischemia in DAB treated-retinae compared to DAB treatment or 30-min ischemia alone (DAB: *n* = 3 retinae; 30 min-ischemia: *n* = 3 retinae; 30-min-ischemia and DAB: *n* = 3 retinae; *P* < 0.001, ANOVA and Tukey’s test). Scale bars in **a**-**e**, **g** = 10 μm

Glial cells use glycogen stores as an alternative source of glucose, which has been proposed to counter toxic intracellular calcium increase during the first hour of ischemia [38, 57–60]. Based on this, we tested whether glycogen stores within glial end-feet at the vascular interface could account for the around 1-h delay in the emergence of ischemia-induced pericyte calcium rise and resultant α-SMA-mediated contraction by providing glucose during compromised glucose transport from blood. Indeed, we observed depletion of glycogen within the perivascular end-feet within 1 h of ischemia induction (Fig. 9a-e). After 1-h ischemia, the number of peri-microvascular glial end-feet displaying low glycogen levels tightly correlated with the presence of constricted microvessels (Pearson product-moment correlation, *R*^*2*^ = 0.992, *P* = 0.0003) (Fig. 9f). Accordingly, a large number of microvessels had low perivascular glycogen in ischemic retinas, whereas control non-ischemic retinas displayed many microvessels surrounded by glial end-feet rich in glycogen. For this analysis, a semiautomatic computer routine was used to stereologically identify lectin-labeled microvessel constrictions and to randomly measure brightness of perivascular PAS staining on a semi-quantitative scale ranging 0 to 1 in increments of 0.2. To further investigate the role of glycogen in pericyte contractions, we intravitreally injected 1,4-Dideoxy-1,4-imino-D-arabinitol hydrochloride (DAB), a potent inhibitor of glycogen phosphorylase, which prevents liberation of glucose from glycogen. DAB caused a striking increase in ischemia-induced capillary constrictions at pericyte locations (Fig. 9g-h). DAB treatment also accelerated the appearance of constrictions such that it doubled the number of constrictions 30 min after ischemia compared to the constrictions at 30 min in ischemic untreated retinas (245 ± 20 vs. 571 ± 29 constrictions, *P* < 0.001, ANOVA Tukey’s test) (Fig. 9h). Collectively, these results suggest that depletion of glycogen in perivascular glial cells can exacerbate pericyte contraction.

## Discussion

We found that ischemia induces constrictions in retinal microvessels similar to those reported for ischemic cortical and coronary microvessels [6, 8, 17]. Our in vivo and ex vivo data show that pericytes were localized at constriction sites*.* In vivo AOSLO imaging also showed that ischemia led to rounding-up of the pericyte somata on microvessels, a typical morphological feature of contracted pericytes [32, 47, 48]. This morphological change was confirmed by ex vivo labeling of pericyte basement membrane ensheating them at the end of in vivo recordings. Constrictions were prevented by knocking down α-SMA expression or by fixing α-SMA with phalloidin [61] or with the L-type channel antagonist amlodipine, suggesting that pericyte contractions are mediated by calcium rise and α-SMA activation. Consistent with this formulation, pericytes located near the microvascular constrictions exhibited significant calcium increase after ischemia as detected by NG2:GCaMP6 or Fluo-4 fluorescence.

Based on reports showing the presence of gap junctions between pericytes and endothelial cells [62], and given that astrocytic gap junctions remain open during ischemia [63], we hypothesized that the source of a fraction of intra-pericytic excess calcium might be the endothelium-pericyte gap junctions in addition to L-type calcium channels. Therefore, we employed the gap junction blocker carbenoxolone and found that it reduced the ischemia-induced calcium rise in pericytes. Unexpectedly, however, this also led to marked calcium rise in the end-feet and soma of adjacent glial cells. We reproduced the same findings with a selective peptide inhibitor of Cx43, confirming that carbenoxolone dose used was sufficient to block gap junctions and calcium signaling. Although elucidation of the underlying mechanisms requires further research, we can speculate that suppression of dissemination of the ischemia-induced ion and water load through the glial syncytium through gap junctions may increase cellular stress and aggravate intraglial calcium rise. Alternatively, these changes in intraglial milieu may directly enhance Fluo-4 fluorescence, which depends on pH and ionic strength; however, similar degrees of enhancement of calcium-induced fluorescence in NG2:GCaMP6 expressing as well as Fluo-4-loaded ischemic pericytes argues against this possibility. These considerations notwithstanding, the observation that Cx43 blockage reduces calcium in pericytes suggests that ischemia-induced calcium signaling (wave) within glia can release vasoconstrictive mediators (e.g. ATP) through connexon (e.g. Cx43) hemichannels clustered at perivascular end-feet overlying pericytes. Retinal ischemia has been shown to increase expression of gap junctions (e.g. connexin 43) in glial cells and capillaries [44, 53, 64]. Rapid induction and clustering of Cx43 at the glia-pericyte interface as observed in our study supports these possibilities and warrants further research. Additionally, increased calcium in depolarized endothelia may trigger calcium signaling by mediators such as inositol trisphosphate propagating to pericytes via gap junctions, and hence, their blockade may also dampen pericyte calcium rise down. Consistent with the reduced calcium rise in pericytes, carbenoxolone also decreased microvessel constrictions during ischemia. Of note, energy deficiency over the course of ischemia is a well-characterized cause of intracellular calcium rise due to enhanced influx, reduced efflux, and impaired intracellular buffering capacity [65]. In addition to two putative influx pathways discussed above, several other factors such as reactive oxygen species (ROS), lactate accumulation and calcium-induced calcium release from endoplasmic reticulum can also contribute to an uncontrolled rise in intracellular calcium [8, 66–69].

Small decreases in capillary radius caused by ischemia-induced pericyte contractions can lead to erythrocyte entrapment because the limited size of capillary lumen requires erythrocyte deformation for an uninterrupted blood flow even under normal physiological conditions [8, 51]. Consistent with this, we found an absence of erythrocyte perfusion in a number of microvessels during ischemia in vivo using AOSLO imaging. Upon recanalization, constricted microvessels were still blocked or poorly reperfused as evidenced by lack of motion contrast in AOSLO movies. The failure of erythrocyte circulation within a fraction of the microvessels and the increased heterogeneity of erythrocyte transit times through slightly constricted capillaries can significantly reduce O_2_ delivery to the tissue [70]. Since adenosine, a powerful pericyte relaxant was shown to reduce erythrocyte entrapments and improve reperfusion in the brain when given during recanalization [71], we also tested whether post-ischemic adenosine treatment could reverse capillary constrictions, hence, may have a therapeutic potential in retinal ischemia as reported for cerebral [71] and coronary ischemia [17]. Adenosine post-treatment indeed reversed ischemia-induced pericyte contractions comparably to its pre-ischemic administration, encouraging that impaired reperfusion (no-reflow) can be treated within the early hours of retinal ischemia before pericytes die in rigor [6]. Of note, our approach to administer reagents into the vitreous chamber not only prevented confounding systemic effects (e.g. of adenosine) and evaded the blood/retinal barrier, but also facilitated access to the retinal interstitium reaching the abluminal side of the vessels and preferentially acting onto receptors and channels on pericytes [72–74].

Interestingly, ischemia-induced pericyte contractions coincided with depletion of glycogen from glial end-feet, suggesting that glycogen, possibly acting as a reservoir of glucose [75, 76], positively contributed to microvascular calcium homeostasis within the first hour of ischemic insult. The observation that inhibition of glycogen breakdown to glucose with DAB accelerated the appearance of microvascular constrictions during ischemia, strongly suggests a role of glycogen in the maintenance of microvascular metabolism during ischemia [38, 57, 58, 60]. These findings are consistent with known resistance of Müller glia to glucose deprivation or hypoxia, which is attributed to their glycogen stores [77]. These observations also warrant further studies to fully elucidate the significance of the metabolic collaboration between glial end-feet and pericytes in the control of microvascular function.

## Conclusions

In conclusion, we showed that disruption of calcium homeostasis in pericytes over the course of the first hour of ischemia caused pericyte contraction mediated by α-SMA. We also showed that peri-microvascular glycogen can delay pericyte contraction by providing glucose to compensate for lack of blood-driven glucose. The observation that pericytes remain contracted after a short period of ischemia and this can pharmacologically be reversed suggest that pericytes could be an attractive target to promote microvessel patency upon vessel occlusion before irreversible injury occurs. Although not investigated in the present study, prevention of irreversible ischemic pericyte damage (e.g. by antioxidants [8]) may additionally reduce plasma exudation and RBC leakage to retina as reported for brain ischemia [78] and cochlear blood-fluid barrier injuries [79] because pericyte dysfunction promotes blood-retina barrier leakiness as well as disruption of the blood-brain and cochlear blood-intrastrial fluid barrier [79, 80]. The observed α-SMA-mediated contractility in response to ischemic injury and the role of calcium in the regulation of the contractile response, refine and expand our understanding of the pericytes in relation to blood flow at the single-capillary level in health as well as neurodegenerative conditions including retinal ischemia, diabetic retinopathy, stroke and Alzheimer’s disease.

## Additional files

Additional file 1: **Figure S1.** Constricted microvascular segments on the ischemic/recanalized whole-mount retinae. Retinae stained with vascular markers claudin-5 (A, C) or lectin (B) illustrate the microvascular constrictions (arrows). These images are the green channel of the merged images in Fig. 2c-e in the main text. (D) Scrambled siRNA injected 24 h before ischemia as a control for α-SMA siRNA did not modify α-SMA expression (red) nor prevent ischemia-induced constrictions (arrows). Vessels were labeled with lectin (green). Scale bar in A-B = 10 μm; scale bar in C = 5 μm; ; in D = 25 μm. (TIF 4329 kb)

Additional file 2: **Figure S2.** Connexin-43 blocking peptide had no effect on intracellular calcium in control non-ischemic retinas. (A, B) Fluo-4 indicator reported no effect on intracellular calcium in control non-ischemic retinas after intravitreal injection of connexin-43 blocking peptide (peptide 5). (B) Vessels were labeled with lectin (red) and nuclei with DAPI (blue). Scale bar in A-B = 5 μm. (TIF 7894 kb)

Additional file 3: **Figure S3.** The action of CBX was not mediated by inhibition of pericytic pannexin-1 channels. One hour after ischemia, pericytes (arrow) were yet not labeled with propidium iodide (PI) **(A)** whereas retinal Hoechst positive parenchyma cells were **(B),** suggesting that the action of CBX at the dose used was not mediated by inhibition of pericytic pannexin-1 channels. Scale bar in A = 10 μm; in B = 50 μm. (TIF 8380 kb)

Additional file 4: **Figure S4.** Pharmacological agents applied did not affect luminal diameter of large vessels in contrast to small capillaries. At the doses used, the pharmacological agents applied intra-vitreally as well as ischemia did not affect luminal diameter of the vessels larger than 9 μm (*P* > 0.05, ANOVA and Tukey’s test). (TIF 2862 kb)

Additional file 12: **Table S1.** Summary of experiments and comparisons. The table summarizes all experiments performed including the treatment groups and number of mice used. Where appropriate, analyses, statistical comparisons, mean values (±SEM), and *P* values are also indicated. Please note that some animals/retinas were used for more than one experiment, therefore, the total number of mice is less than total number of experiments. (DOCX 45 kb)

## Abbreviations

α-SMA: alpha-smooth muscle actin
ATP: Adenosine triphosphate
AOSLO: Adaptive optics scanning light ophthalmoscopy
Asf: Area-sampling fraction
ANOVA: Analysis of Variance
a.u: Arbitrary units
Cx43: Connexin 43
Ca2+: Calcium
CRALBP: Cellular retinaldehyde binding protein
CBX: Carbenoxolone
DAB: 1,4-Dideoxy-1,4-imino-D-arabinitol hydrochloride
F-actin: Filamentous actin
FITC: Fluorescein Isothiocyanate
IHC: immunohistochemistry
LSC: Laser speck contrast
NG2: Neural glial antigen-2
NIR: Near-infrared
RNA: Ribonucleic acid
RBF: Pseudocolored relative blood flow
PFA: Paraformaldehyde
PBS: 1X phosphate buffer solution
PBST: PBS containing 0.5% Triton X-100
PDGFRβ: Platelet-derived growth factor receptor beta
PAS: Periodic acid-Schiff
PMT: Photomultiplier tube
PI: Propidium iodide
ROI: Region of interest
ROS: Reactive oxygen species
Ssf: Section-sampling fraction
siRNA: Short interfering RNA
tPA: Tissue plasminogen activator
TRITC: Tetramethylrhodamine
Tsf: Thick-sampling fraction
