# Supplementary figures and images for: Retinal ischemia induces α-SMA-mediated capillary pericyte contraction coincident with perivascular glycogen depletion
Source: Acta Neuropathol Commun. 2019 Aug 20;7:134. doi: 10.1186/s40478-019-0761-z (PMC6701129; doi:10.1186/s40478-019-0761-z)

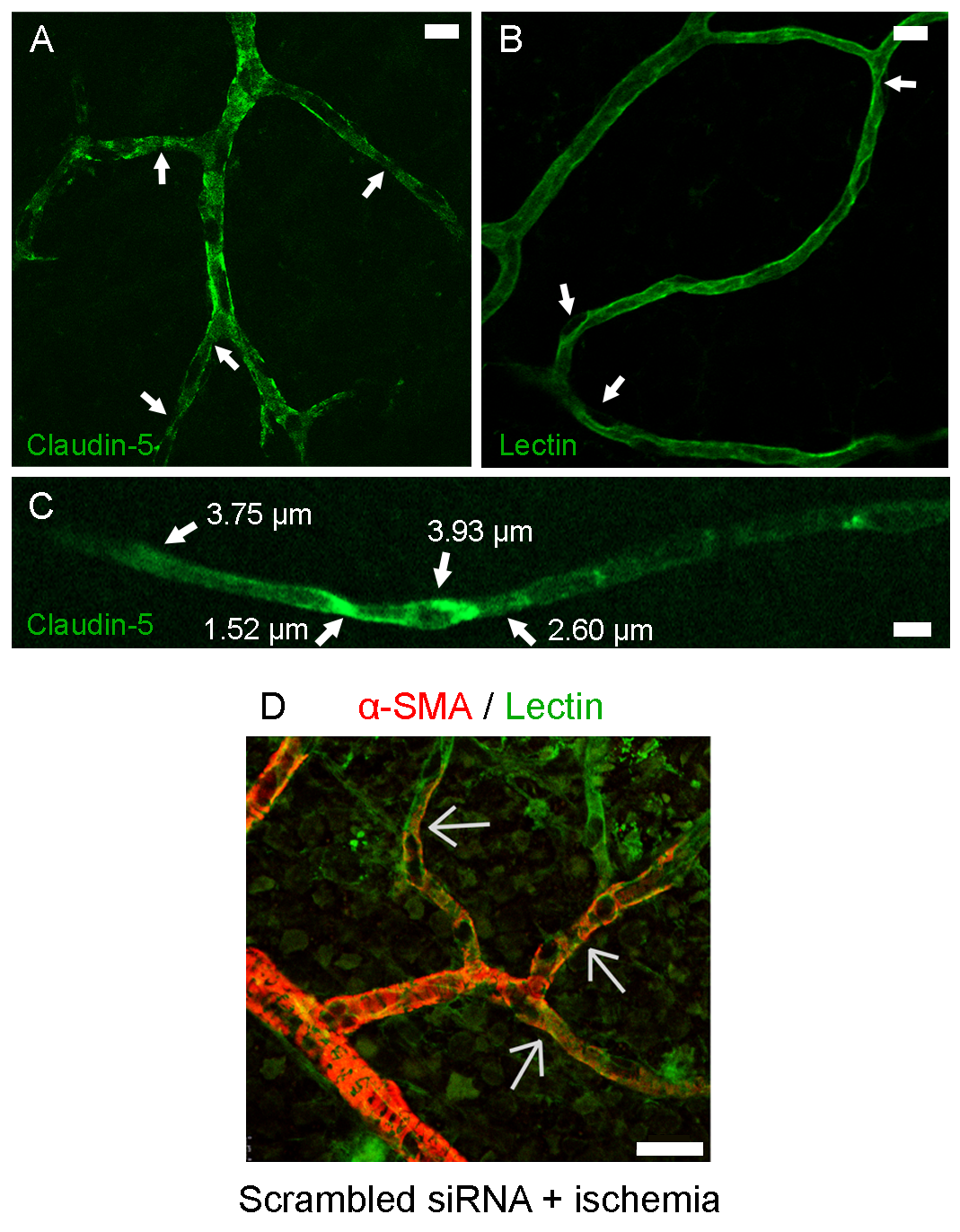

Supplement: Supplementary file 8 — Figure S1. Constricted microvascular segments on the ischemic/recanalized whole-mount retinae. Retinae stained with vascular markers claudin-5 (A, C) or lectin (B) illustrate the microvascular constrictions (arrows). These images are the green channel of the merged images in Fig. 2c-e in the main text. (D) Scrambled siRNA injected 24 h before ischemia as a control for α-SMA siRNA did not modify α-SMA expression (red) nor prevent ischemia-induced constrictions (arrows). Vessels were labeled with lectin (green). Scale bar in A-B = 10 μm; scale bar in C = 5 μm; ; in D = 25 μm. (TIF 4329 kb) [file 40478_2019_761_MOESM1_ESM.tif]

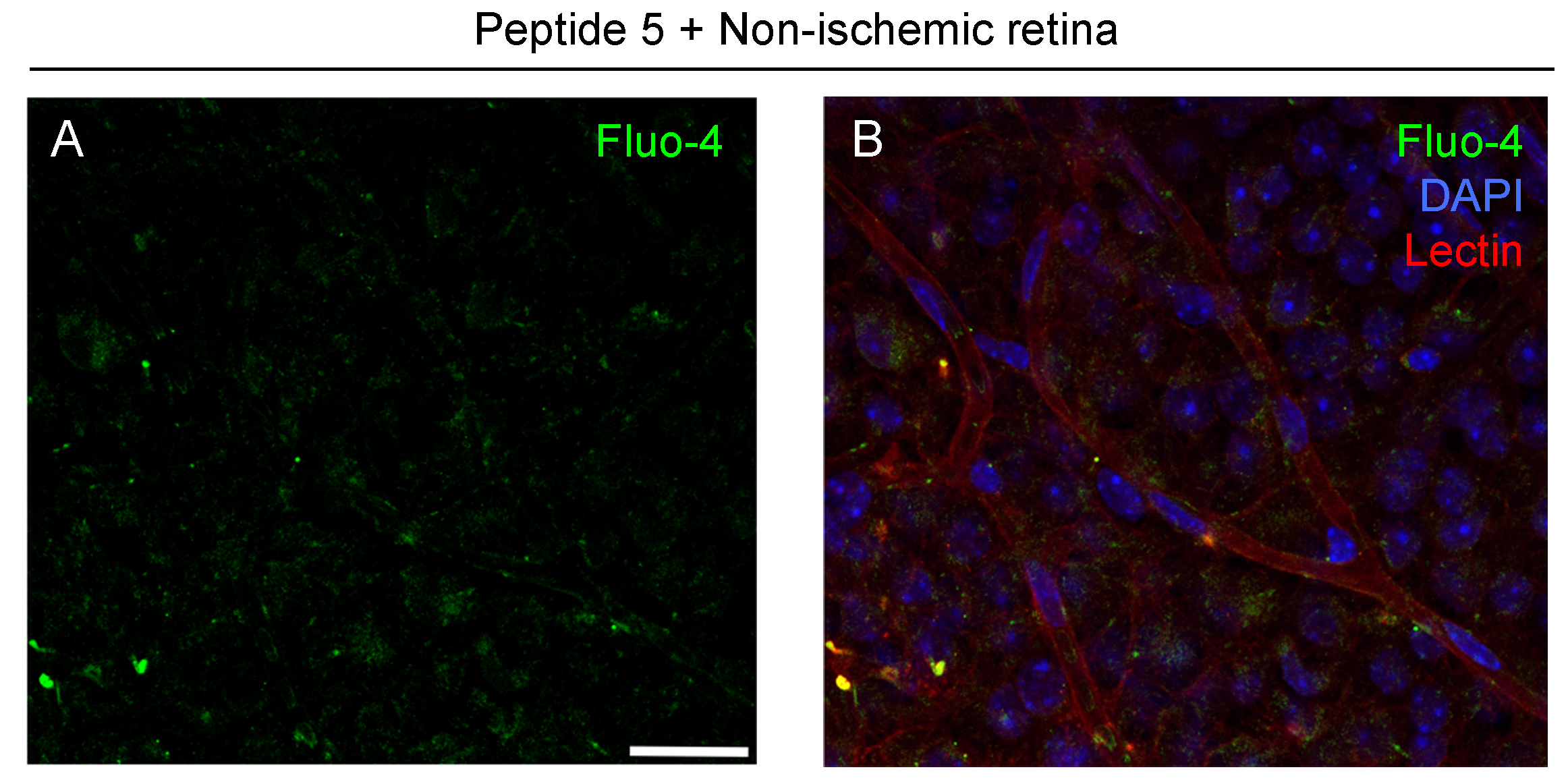

Supplement: Supplementary file 9 — Figure S2. Connexin-43 blocking peptide had no effect on intracellular calcium in control non-ischemic retinas. (A, B) Fluo-4 indicator reported no effect on intracellular calcium in control non-ischemic retinas after intravitreal injection of connexin-43 blocking peptide (peptide 5). (B) Vessels were labeled with lectin (red) and nuclei with DAPI (blue). Scale bar in A-B = 5 μm. (TIF 7894 kb) [file 40478_2019_761_MOESM2_ESM.tif]

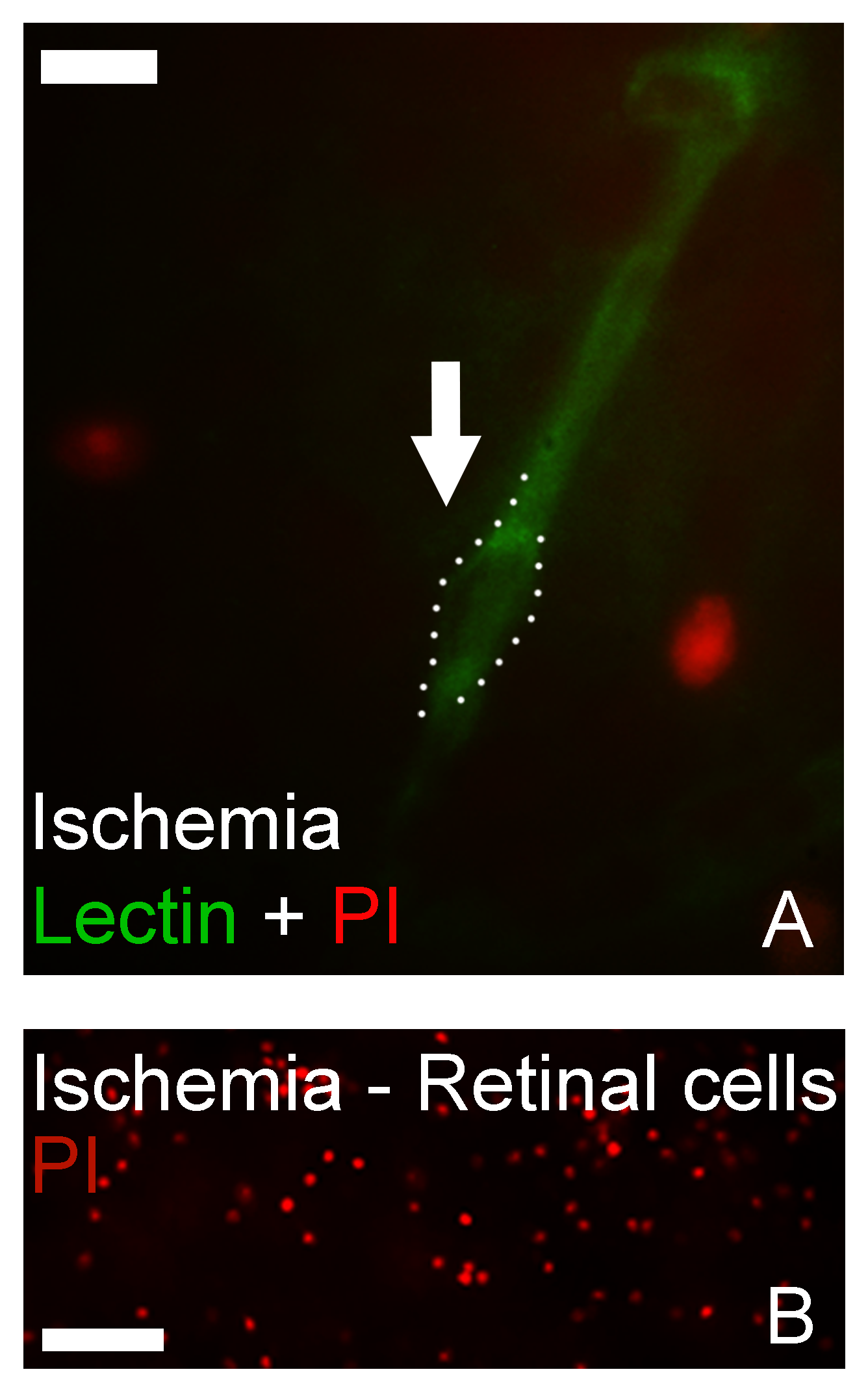

Supplement: Supplementary file 10 — Figure S3. The action of CBX was not mediated by inhibition of pericytic pannexin-1 channels. One hour after ischemia, pericytes (arrow) were yet not labeled with propidium iodide (PI) (A) whereas retinal Hoechst positive parenchyma cells were (B), suggesting that the action of CBX at the dose used was not mediated by inhibition of pericytic pannexin-1 channels. Scale bar in A = 10 μm; in B = 50 μm. (TIF 8380 kb) [file 40478_2019_761_MOESM3_ESM.tif]

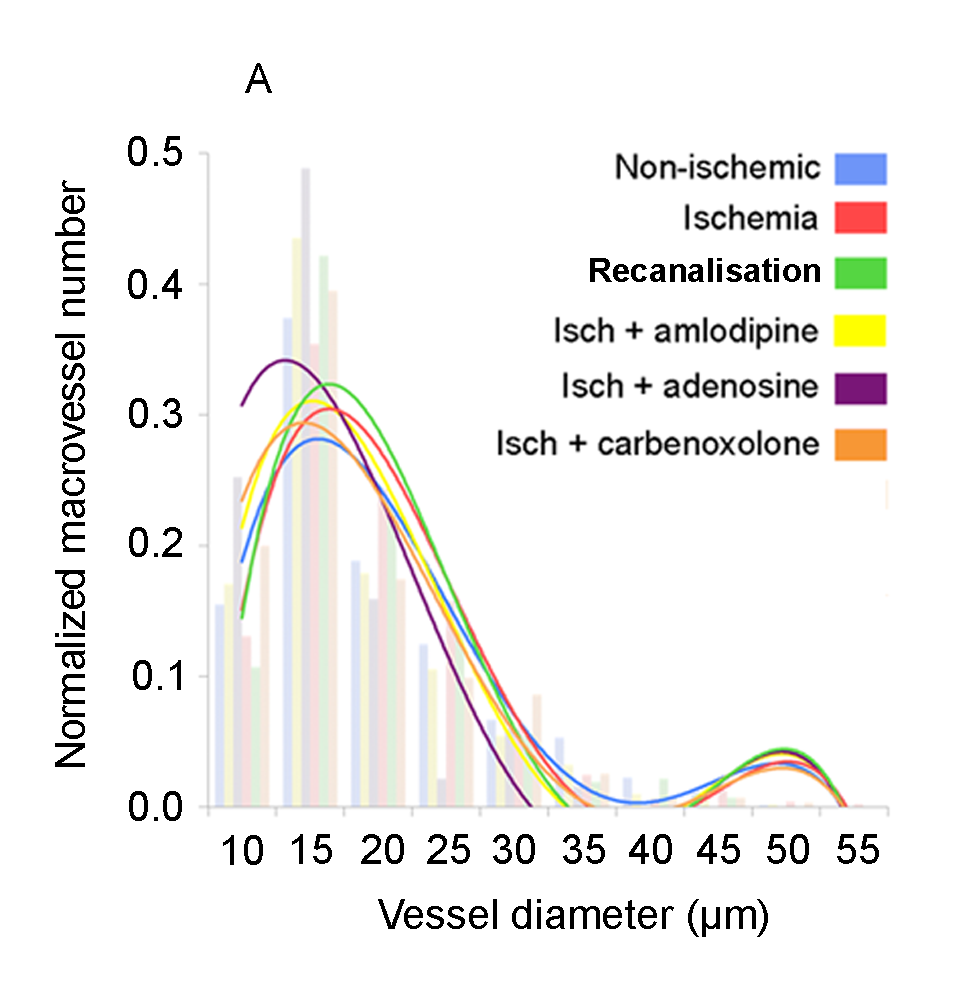

Supplement: Supplementary file 11 — Figure S4. Pharmacological agents applied did not affect luminal diameter of large vessels in contrast to small capillaries. At the doses used, the pharmacological agents applied intra-vitreally as well as ischemia did not affect luminal diameter of the vessels larger than 9 μm (P > 0.05, ANOVA and Tukey’s test). (TIF 2862 kb) [file 40478_2019_761_MOESM4_ESM.tif]
