# Supplementary material for: Retinal ischemia induces α-SMA-mediated capillary pericyte contraction coincident with perivascular glycogen depletion
Source: Acta Neuropathol Commun. 2019 Aug 20;7:134. doi: 10.1186/s40478-019-0761-z (PMC6701129; doi:10.1186/s40478-019-0761-z)
Supplement: Supplementary file 12 — Table S1. Summary of experiments and comparisons. The table summarizes all experiments performed including the treatment groups and number of mice used. Where appropriate, analyses, statistical comparisons, mean values (±SEM), and P values are also indicated. Please note that some animals/retinas were used for more than one experiment, therefore, the total number of mice is less than total number of experiments. (DOCX 45 kb) [file 40478_2019_761_MOESM12_ESM.docx]

| **Experiment** | | **Group** | | **n of mice** | |  |
| --- | --- | --- | --- | --- | --- | --- |
| Establishment of ischemia/recanalisation model | | Vitreous hemorrhage (excluded) | | 5 | |  |
|  |  | 10%-FeCl3 ischemia | | 3 | |  |
|  |  | 20%-FeCl3 ischemia | | 13 | |  |
|  |  | 30%-FeCl3 ischemia | | 7 | |  |
|  |  | 30%-FeCl3 ischemia and tPA | | 4 | |  |
| Ischemia/recanalisation surgery | | Ischemia (20%-FeCl3) | | 66 | |  |
|  |  | Recanalisation (tPA) | | 9 | |  |
|  |  | Ischemia/recanalisation (suture) | | 20 | |  |
|  |  | Non-retina ischemia due to poor contact | | 18 | |  |
| Laser-speckle flowmetry | | Ischemia | | 67 | |  |
|  |  | Recanalisation | | 8 | |  |
|  |  | Non-retinal ischemia due to poor contact | | 18 | |  |
|  |  | Establishment of model | | 27 | |  |
| In vivo fluorescence retinal angiography | | Non-ischemia (contralateral) | | 14 | |  |
|  |  | Ischemia | | 14 | |  |
|  |  | Recanalisation | | 6 | |  |
| Ex vivo fluorescence imaging of vasculature | | Non-ischemia (contralateral) | | 10 | |  |
|  |  | Ischemia | | 6 | |  |
|  |  | Recanalisation | | 6 | |  |
| Ex vivo NG2-DsRed retinas observation | | Non-ischemia (contralateral) | | 3 | |  |
|  |  | Ischemia/recanalisation | | 3 | |  |
| Ex vivo constrictions localized by lectin + PDGFRb labeling | | Ischemia | | 4 | |  |
| In vivo propidium iodide injection | | Non-ischemia (contralateral) | | 2 | |  |
|  |  | Ischemia + PI | | 3 | |  |
| Calcium colocalization | | In vivo fluo-4 injection in ischemic DsRed mice | | 4 | |  |
| **Experiment** | **Analysis** | **Comparison** | **Intensity (mean±SEM)** | **n of mice (ROIs)** | **P** |  |
| **In vivo fluorescence retinal angiography** | **In vivo intensity of retinal tissue (i.e. area covered by microvessels)** | **Non-ischemia vs. ischemia** | 114.7 ± 9.07 (a.u.) vs. 39.6 ± 2.24 (a.u.) | 3 (36) vs. 4 (40) | <0.0001*  (ANOVA) |  |
|  |  | **Non-ischemia vs. recanalisation** | 114.7 ± 9.07 (a.u.) vs. 50.83 ± 2.66 (a.u.) | 3 (36) vs. 4 (60) | <0.0001*  (ANOVA) |  |
|  |  | **Ischemia vs. recanalisation** | 39.6 ± 2.24 (a.u.) vs. 50.83 ± 2.66 (a.u.) | 4 (40) vs. 4 (60) | n.s.  (ANOVA) |  |
| **Experiment** | **Analysis** | **Comparison** | **Diameter (mean±SEM)** | **n of mice (number of microvessels)** | **P** |  |
| **Ex vivo whole-mount retina stereology** | **Ex vivo microvessel diameter** | **Non-ischemia vs. ischemia** | 4.772 ± 0.041 μm vs. 4.412 ± 0.221 μm | 3 (4531) vs. 3 (4268) | <0.0001*  (ANOVA) |  |
|  |  | **Non-ischemia vs. recanalisation** | 4.772 ± 0.041 μm vs. 4.156 ± 0.164 μm | 3 (4531) vs. 3(5444) | <0.0001*  (ANOVA) |  |
|  |  | **Ischemia vs. recanalisation** | 4.412 ± 0.221 μm vs. 4.156 ± 0.164 μm | 3 (4268) vs. 3 (5444) | <0.0001*  (ANOVA) |  |
|  |  | **Phalloidin vs. non-ischemia** | 4.698 ± 0.200 μm vs. 4.772 ± 0.041 μm | 3 (1993) vs. 3(4531) | n.s.  (ANOVA) |  |
|  |  | **Phalloidin vs. ischemia** | 4.698 ± 0.200μm vs. 4.412 ± 0.221 μm | 3 (1993) vs. 3 (4268) | <0.0001*  (ANOVA) |  |
|  |  | **Phalloidin vs. recanalisation** | 4.698 ± 0.200 μm vs. 4.156 ± 0.164 μm | 3 (1993) vs. 3 (5444) | <0.0001*  (ANOVA) |  |
|  |  | **Mixed-effect model analysis** | | | <0.0001* |  |
|  | **Analysis** | **Comparison** | **Number of constrictions (mean±SEM)** | **n of mice** | **P** |  |
|  | **Ex vivo number of microvessel constrictions co-localized with NG2 pericytes** | **Non-ischemia vs. ischemia** | 48.4 ± 10.15 vs. 146.7 ± 26.64 | 5 vs. 3 | 0.004*  (ANOVA) |  |
|  |  | **Non-ischemia vs. recanalisation** | 48.4 ± 10.15 vs. 144.9 ± 15.33 | 5 vs. 5 | 0.002*  (ANOVA) |  |
|  |  | **Mixed-effect model analysis** | | | 0.045* |  |
|  | **Analysis** | **Comparison** | **Percent of constrictions (mean±SEM)** | **n of mice** | **P** |  |
|  | **Ex vivo number of microvessel constrictions – junctional and helical** | **Junctional constrictions vs. helical constrictions** | 63.37 ± 4.54 % vs.  48.19 ± 2.91% | 3 | 0.006  (*t*-test) |  |
| **Experiment** | **Analysis** | **Comparison** | **Diameter (mean±SEM)** | **n of mice (number of microvessels)** | **P** |  |
| **AOSLO**  **(in vivo)** | **In vivo microvascular diameter** | **Pre-ischemia vs. recanalisation** | 3.527 ± 0.127 μm vs. 2.857 ± 0.065 μm | 4 (143) vs. 3 (441) | <0.0001*  (t-test) |  |
|  | **Analysis** | **Comparison** | **Intensity (mean±SEM) (soma length)** | **n of mice (number of pericytes)** | **P** |  |
|  | **In vivo pericyte shape (soma)** | **Pre-ischemia vs. ischemia 0-1h** | 0.463 ± 0.024 (4.3 μm) vs. 0.514 ± 0.014 (4.7 μm) | 4 (72) vs. 3 (226) | n.s.  (ANOVA) |  |
|  |  | **Pre-ischemia vs. ischemia 1-2h** | 0.445 ± 0.024 (4.3 μm) vs. 0.545 ± 0.018 (5.5 μm) | 4 (72) vs. 4 (125) | 0.0005*  (ANOVA) |  |
|  |  | **Pre-ischemia vs. recanalisation** | 0.400 ± 0.022 (4.3 μm) vs. 0.502 ± 0.009 (5.9 μm) | 4 (72) vs. 3 (530) | 0.005*  (ANOVA) |  |
|  |  | **Ischemia 0-1h vs. ischemia 1-2h** | 0.477 ± 0.014 (4.7 μm) vs. 0.544 ± 0.018 (5.5 μm) | 3 (226) vs. 4 (125) | 0.004*  (ANOVA) |  |
|  |  | **Ischemia 0-1h vs. recanalisation** | 0.434 ± 0.013 (4.7 μm) vs. 0.502 ± 0.009 (5.9 μm) | 3 (226) vs. 3 (530) | 0.036*  (ANOVA) |  |
|  |  | **Ischemia 1-2h vs. recanalisation** | 0.492 ± 0.018 (5.5 μm) vs. 0.502 ± 0.009 (5.9 μm) | 4 (125) vs. 3 (530) | n.s.  (ANOVA) |  |
|  |  | **Mixed-effect model analysis** | | | <0.0001* |  |
| **Pericyte morphological analysis**  ***(*ex vivo*)*** | **Analysis** | **Comparison** | **Shape factor (mean±SEM)** | **n of mice (number of pericytes)** | **P** |  |
|  | **Pericyte *basement membrane* morphology after labeling by lectin** | **Pre-ischemia vs. ischemia 1h** | 1.25 ± 0.060 vs. 1.00 ± 0.020 | 3 (530) vs. 3 (655) | 0.006*  (ANOVA) |  |
|  |  | **Pre-ischemia vs. recanalisation** | 1.25 ± 0.060 vs. 1.03 ± 0.016 | 3 (530) vs. 3 (387) | 0.01*  (ANOVA) |  |
|  |  | **Mixed-effect model analysis** | | | 0.001* |  |
| **Experiment** | **Analysis** | **Comparison** | **Number of cells (mean±SEM)** | **n of mice** | **P** |  |
| **Ex vivo whole-mount retina stereology: mechanisms of ischemia-induced pericyte contractions.** | **In vivo fluo-4 injection: number of calcium-loaded pericytes** | **Non-ischemia vs. ischemia** | 183 ± 25.6 vs. 605 ± 159.0 | 7 vs. 3 | 0.001*  (ANOVA) |  |
|  |  | **Ischemia vs. ischemia + CBX** | 605 ± 159.0 vs. 119 ± 35.7 | 3 vs. 4 | 0.018*  (ANOVA) |  |
|  |  | **Mixed-effect model analysis** | | | 0.026* |  |
|  | **In vivo fluo-4 injection: number of glia-like cells** | **Non-ischemia vs. ischemia + CBX** | 2.97 ± 1.76 vs. 50.42 ± 9.03 | 7 vs. 4 | <0.0001*  (ANOVA) |  |
|  |  | **Ischemia vs. ischemia + CBX** | 1.35 ± 1.35 vs. 50.42 ± 9.03 | 3 vs. 4 | <0.0001*  (ANOVA) |  |
|  |  | **Mixed-effect model analysis** | | | 0.011* |  |
|  | **In vivo NG2:GCaMP6: number of calcium-loaded pericytes** | **Non-ischemia vs. ischemia** | 72.47 ± 59.47 vs. 804.00 ± 199.72 | 5 vs. 3 | 0.001*  (ANOVA) |  |
|  |  | **Ischemia vs. ischemia + CBX** | 804.00 ± 199.72 vs. 345.60 ± 71.64 | 3 vs. 5 | 0.034*  (ANOVA) |  |
|  |  | **Mixed-effect model analysis** | | | <0.008* |  |
|  | **Analysis** | **Comparison** | **Intensity (mean±SEM)** | **n of mice** | **P** |  |
|  | **In vivo NG2:GCaMP6: intracellular calcium in pericytes** | **Non-ischemia vs. 40 min ischemia** | 49.67 ± 11.09 (a.u.) vs. 194.63 ± 17.01 (a.u.) | 4 vs. 3 | <0.0001*  (ANOVA) |  |
|  |  | **Non-ischemia vs. 60 min ischemia** | 49.67 ± 11.09 (a.u.) vs. 211.87 ± 21.28 (a.u.) | 4 vs. 3 | <0.0001*  (ANOVA) |  |
|  |  | **Non-ischemia vs. 60 min ischemia + CBX** | 49.67 ± 11.09 (a.u.) vs. 125.72 ± 13.39 (a.u.) | 4 vs. 5 | 0.003*  (ANOVA) |  |
|  |  | **60 min ischemia + CBX vs. 40 min ischemia** | 125.72 ± 13.39 (a.u.) vs. 194.63 ± 17.01 (a.u.) | 5 vs. 3 | 0.007*  (ANOVA) |  |
|  |  | **60 min ischemia + CBX vs. 60 min ischemia** | 125.72 ± 13.39 (a.u.) vs. 211.87 ± 21.28 (a.u.) | 5 vs. 3 | 0.002*  (ANOVA) |  |
|  |  | **Mixed-effect model analysis** | | | <0.0001* |  |
|  | **Analysis** | **Comparison** | **Number of constrictions (mean±SEM)** | **n of mice** | **P** |  |
|  | **Ex vivo number of microvessel constrictions** | **Non-ischemia vs. ischemia** | 104 ± 10.4 vs. 269 ± 33.9 | 5 vs. 3 | 0.005*  (ANOVA) |  |
|  |  | **Ischemia vs. recanalisation** | 269 ± 33.9 vs. 305 ± 57.5 | 3 vs. 3 | n.s.  (ANOVA) |  |
|  |  | **Ischemia vs. adenosine (prior to ischemia)** | 269 ± 33.9 vs. 155 ± 13.8 | 3 vs. 4 | 0.010*  (ANOVA) |  |
|  |  | **Ischemia vs. adenosine (1h after ischemia)** | 269 ± 33.9 vs. 155 ± 33.7 | 3 vs. 3 | 0.020*  (ANOVA) |  |
|  |  | **Ischemia vs. ischemia + amlodipine** | 269 ± 33.9 vs. 104 ± 21.9 | 3 vs. 3 | 0.001*  (ANOVA) |  |
|  |  | **Ischemia vs. ischemia + CBX** | 269 ± 33.9 vs. 148 ± 3.6 | 3 vs. 4 | 0.007*  (ANOVA) |  |
|  |  | **Ischemia vs. ischemia + phalloidin** | 269 ± 33.9 vs. 117 ± 21.1 | 3 vs. 5 | <0.001*  (ANOVA) |  |
|  |  | **Recanalisation vs. adenosine (prior to ischemia)** | 305 ± 57.5 vs. 155 ± 13.8 | 3 vs. 4 | 0.005*  (ANOVA) |  |
|  |  | **Recanalisation vs. adenosine (1h after ischemia)** | 305 ± 57.5 vs. 155 ± 33.7 | 3 vs. 3 | 0.009*  (ANOVA) |  |
|  |  | **Recanalisation vs. ischemia + amlodipine** | 305 ± 57.5 vs. 104 ± 21.9 | 3 vs. 3 | <0.001*  (ANOVA) |  |
|  |  | **Recanalisation vs. ischemia + CBX** | 305 ± 57.5 vs. 148 ± 3.6 | 3 vs. 4 | 0.004*  (ANOVA) |  |
|  |  | **Recanalisation vs. ischemia + phalloidin** | 305 ± 57.5 vs. 117 ± 21.1 | 3 vs. 5 | <0.001*  (ANOVA) |  |
|  |  | **Mixed-effect model analysis** | | | 0.049* |  |
|  | **Ex vivo diameter of microvessel (< 7 μm) (siRNA)** | **Scrambled siRNA + ischemia vs. α-SMA siRNA + ischemia** | 4.076 ± 0.121 μm vs. 4.521 ± 0.121 μm | 3 vs. 3 | 0.01*  (*t-*test) |  |
|  | **Ex vivo number of microvessel constrictions (siRNA)** | **Scrambled siRNA + ischemia vs. non-ischemia** | 291 ± 24.4 vs. 139 ± 8.2 | 3 vs. 3 | 0.007*  (ANOVA) |  |
|  |  | **Scrambled siRNA + ischemia vs. α-SMA siRNA + ischemia** | 291 ± 24.4 vs. 138 ± 28.8 | 3 vs. 3 | 0.007*  (ANOVA) |  |
|  |  | **Mixed-effect model analysis** | | | 0.036* |  |
|  | **Analysis** | **Comparison** | **Diameter (mean±SEM)** | **n of mice (number of macrovessels)** | **P** |  |
|  | **Ex vivo macrovascular diameter** | **Non-ischemia vs. ischemia** | 16.9 ± 0.82 μm vs. 16.9 ± 0.84 μm | 3 (655) vs. 3 (443) | n.s.  (ANOVA) |  |
|  |  | **Non-ischemia vs. recanalisation** | 16.9 ± 0.82 μm vs. 16.3 ± 0.29 μm | 3 (655) vs. 3 (410) | n.s.  (ANOVA) |  |
|  |  | **Ischemia vs. recanalisation** | 16.9 ± 0.84 μm vs. 16.3 ± 0.29 μm | 3 (443) vs. 3 (410) | n.s.  (ANOVA) |  |
|  |  | **Ischemia vs. ischemia + adenosine** | 16.9 ± 0.84 μm vs. 14.0 ± 0.71 μm | 3 (443) vs. 3 (470) | n.s.  (ANOVA) |  |
|  |  | **Ischemia vs. ischemia + amlodipine** | 16.9 ± 0.84 μm vs. 15.6 ± 0.06 μm | 3 (443) vs. 3 (491) | n.s.  (ANOVA) |  |
|  |  | **Ischemia vs. ischemia + CBX** | 16.9 ± 0.84 μm vs. 15.7 ± 0.38 μm | 3 (443) vs. 3 (544) | n.s.  (ANOVA) |  |
|  |  | **Mixed-effect model analysis** | | | 0.153 |  |
| **Experiment** | **Analysis** | **Comparison** | **Area (mean±SEM)** | **n of mice (number of microvessels)** | **P** |  |
| **Pericapillary endfeet area exhibiting Cx43** | **3D analysis** | **Non-ischemia vs. ischemia** | 20.65 ± 5.46 μm^2^ vs. 337.32 ± 133.57 μm^2^ | 3 (18) vs. 4 (15) | 0.014  (*t*-test) |  |
| **Experiment** | **Analysis** | **Comparison** | **Brightness (mean±SEM)** | **n of mice (number of microvessels)** | **P** |  |
| **Ex vivo semiautomatic glycogen measurement in microvessel glial end-feet** | **Total glycogen amount** | **Non-ischemia vs. ischemia** | 17.6 ± 0.15 vs. 19.10 ± 0.15 | 3 (1139) vs. 3 (964) | <0.0001*  (t-test) |  |
|  | **Analysis** | **Correlation** | **Ratio** | **n of mice (number of microvessels; constrictions)** | **Pearson test** |  |
|  | **Constrictions ratio (ischemia/non-ischemia) according to glycogen levels** | **Constrictions ratio** | 424% vs. 0.2; 302% vs. 0.4; 202% vs. 0.6; 131% vs. 0.8; 0% vs. 1.0 | 3 non-ischemia (14777; 481) vs. 3 ischemia (12627; 1206) | P<0.0001; R^2^ = 0.992* |  |
| **Ex vivo whole-mount retina stereology: mechanisms of ischemia-induced pericyte contractions** | **Analysis** | **Comparison** | **Number of constrictions (mean±SEM)** | **n of mice** | **P** |  |
|  | **Ex vivo number of microvessel constrictions *after* in vivo glycogen degradation prevention by DAB** | **Non-ischemia + DAB vs. 30 minutes ischemia** | 224 ± 29.6 vs. 245 ± 20.0 | 3 vs. 3 | n.s.  (ANOVA) |  |
|  |  | **Non-ischemia + DAB vs. DAB + 30 minutes ischemia** | 224 ± 29.6 vs. 571 ± 29.0 | 3 vs. 3 | <0.0001*  (ANOVA) |  |
|  |  | **30 minutes ischemia vs. DAB + 30 minutes ischemia** | 245 ± 20.0 vs. 571 ± 29.0 | 3 vs. 3 | <0.0001*  (ANOVA) |  |
|  |  | **Mixed-effect model analysis** | | | 0.003* |  |
|  |  |  |  |  |  |  |

**Table S1. Summary of experiments and comparisons.** The table summarizes all experiments performed including the treatment groups and number of mice used. Where appropriate, analyses, statistical comparisons, mean values (±SEM), and P values are also indicated. Please note that some animals/retinas were used for more than one experiment, therefore, the total number of mice is less than total number of experiments.
